# Characterization of longitudinal nasopharyngeal microbiome patterns in maternally HIV-exposed Zambian infants

**DOI:** 10.12688/gatesopenres.14041.1

**Published:** 2022-11-11

**Authors:** Aubrey R. Odom, Christopher J. Gill, Rachel Pieciak, Arshad Ismail, Donald Thea, William B. MacLeod, W. Evan Johnson, Rotem Lapidot

**Affiliations:** 1 Bioinformatics Program, Boston University, Boston, MA, 02118, USA; 2 Department of Global Health, Boston University School of Public Health, Boston, MA, 02118, USA; 3 Sequencing Core Facility, National Institute for Communicable Diseases of the National Health Laboratory Service, 2131 Johannesburg, South Africa; 4 Department of Biochemistry and Microbiology, University of Venda, Thohoyandou 0950, South Africa; 5 Division of Infectious Disease, Center for Data Science, Rutgers New Jersey Medical School, Newark, NJ, 07103, USA; 6 Pediatric Infectious Diseases, Boston Medical Center, Boston, MA, 02118, USA; 7 Department of Pediatrics, Boston University School of Medicine, Boston, MA, 02118, USA

**Keywords:** nasopharyngeal microbiome, longitudinal cohort study, microbial communities, HIV exposure, children

## Abstract

**Background:** Previous studies of infants born to HIV-positive mothers have linked HIV exposure to poor outcomes from gastrointestinal and respiratory illnesses, and to overall increased mortality rates. The mechanism behind this is unknown, but it is possible that differences in the nasopharyngeal (NP) microbiome between HIV-unexposed and HIV-exposed infants could play a role in perpetuating some outcomes.

**Methods:** We conducted a longitudinal analysis of 170 NP swabs of healthy HIV-exposed, uninfected (HEU; n=10) infants and their HIV(+) mothers and HIV-unexposed, uninfected (HUU; n=10) infants and their HIV(-) mothers. These swabs were identified from a sample library collected in Lusaka, Zambia between 2015 and 2016. Using 16S rRNA gene sequencing, we characterized the maturation of the microbiome over the first 14 weeks of life to determine what quantifiable differences exist between HEU and HUU infants, and what patterns are reflected in the mothers' NP microbiomes.

**Results:** In both HEU and HUU infants, *Staphylococcus* and *Corynebacterium* began as primary colonizers of the NP microbiome but were in time replaced by *Dolosigranulum*, *Streptococcus*, *Moraxella* and *Haemophilus*. When studying differences between infants, the microbe *Staphylococcus haemolyticus* indicated a distinctive high association with HIV exposure at birth, even when accounting for the interaction between HIV exposure status and time of sampling. When comparing infants to their mothers with paired analyses, HEU infants’ NP microbiome composition was only slightly different from their HIV(+) mothers at birth or 14 weeks, including in their carriage of *S. pneumoniae*, *H. influenzae*, and *S. haemolyticus*.

**Conclusions:** Our analyses indicate that the HEU infants in our study exhibit subtle differences in the NP microbial composition throughout the sampling interval. Given our results and the sampling limitations of our study, we believe that further research must be conducted in order to confidently understand the relationship between HIV exposure and infants’ NP microbiomes.

## Introduction

Currently, more than one million infants are born to HIV-infected [HIV(+)] women worldwide every year. ^1^ Fortunately, with antiretroviral treatment for mothers and prophylaxis for their infants, the vast majority of HIV-exposed infants will not become infected with HIV. ^2^ However, prevention of mother-to-child transmission (PMTCT) does not eradicate health disparities between HIV-exposed, uninfected (HEU) and HIV-unexposed, uninfected (HUU) infants by eliminating HIV transmission. Data suggests these children are still directly or indirectly affected by their mother’s HIV status. For example, recent meta-analyses published by members of our team reported a 60% increased risk of death ^3^ and an increased risk of pneumonia and diarrhea ^4^ among HEU compared with HUU children, thereby supporting the observed phenomenon that infants are vulnerable not only to morbidity, but also to increased mortality. These findings have also been observed in numerous other studies that have shown the linkage between poor outcomes and gastrointestinal and respiratory illnesses. ^5– 9^ Hypothesized explanations include dysregulation of passive immunity via maternal antibodies, changes in the maturation of infant lymphocytes, exposure to microbes present in the mother’s birth canal at delivery, and/or social factors, which may have an impact on morbidity and mortality rates in the early stages of life.

Alternatively, it remains possible that many of these reported health differences are merely artifacts of various selection biases. Previous studies on HIV exposure phenomenon were cross sectional studies based on convenience sampling and/or lacked precision due to small sample sizes. ^3, 4^ Further, few such studies were longitudinal, making it difficult to observe changes over time. ^3, 4^ When studying the microbiome, integral to the early development of the immune system, the lack of longitudinal structure is a crucial limitation given that it evolves dynamically over the first days and weeks of life, as both we and others have previously demonstrated. ^10, 11^ Thus, it remains possible that much of the HEU phenomenon as currently described could be due to sociological factors, or simply selection bias. To better understand the potential biological basis for this phenomenon, unbiased, systematic data are required to build confidence in its existence.

In the gut, interactions between the microbial community and the host influence the development of the immune system and, consequently, the development of diseases. ^12^ While much is known about the evolution of the intestinal microbiome, far less is known about the dynamics surrounding the microbiome of the upper respiratory tract, which plays an important role in respiratory health. If the increased rates of respiratory disease observed among HEU children have a biological explanation, we hypothesized that the respiratory microbiomes of HEU infants would differ systematically from HUU infants over the first few months of life, potentially explaining their greater susceptibility to certain respiratory diseases. Previous studies on the impact of HIV exposure have highlighted strong correlations between HIV exposure and increased risk of pneumococcal colonization and disease, ^7, 13, 14^ whereas others have demonstrated no differences in pathogen carriage between children with HIV infection and control groups. ^15– 17^ The current analysis seeks to address this important knowledge gap.

Given the complex dynamics of interactions between the host, microbes, and environment beginning at birth, we conducted an exploratory longitudinal comparison of the nasopharyngeal (NP) microbiomes of HEU and HUU infants. We reasoned that this pilot study could provide insight into the presence or absence of immunological factors distinguishing these two groups. We collected 167 samples from 10 HEU and 10 HUU infants and their mothers from Lusaka, Zambia, which were obtained shortly after the infants’ birth and at two-week intervals thereafter through 14 weeks of life. We sought to characterize the NP microbiome of these infants to determine what quantifiable differences exist between HEU and HUU infants.

## Methods

A subset of 170 NP swabs of 20 infants and their mothers were identified from a sample library collected in Lusaka, Zambia between 2015 and 2016. The sample library was part of a nested time-series case comparator study within the prospective longitudinal Southern Africa Mother-Infant Pertussis study (SAMIPS). ^18^ For the cohort, infants were included if they were otherwise ‘healthy’ when screened at one week of age. Healthy infants were born at term (>37 weeks); not underweight (>2500 grams); had no acute or chronic conditions known at the time of enrollment; were not born via cesarean section; and had no known complications during pregnancy or labor and delivery.

The institutional review boards at Boston Medical Center and Excellence in Research Ethics and Science Converge in Lusaka jointly provided ethical oversight (The ERES Converge, Lusaka. REF# 2015-Jan-002, Date: 01/02/2015; BUMC IRB, Boston. # H-33521, Date: 12/12/2014). All mothers provided written informed consent, with consent forms presented in English and the two dominant vernacular languages spoken in Lusaka: Bemba and Nyanja.The present analysis uses HEU ( *n*=10) infants and their HIV(+) mothers alongside HUU healthy control ( *n*=10) infants and their HIV-negative [HIV(-)] mothers collected as part of the SAMIPS study. Characteristics of the study cohort are delineated in Table 1. Mother-infant pairs were recruited during their first scheduled postpartum well-child visit at approximately one week of age. Infants and their mothers were enrolled from the Chawama Primary Health Clinic (PHC) in Chawama compound, a densely populated peri-urban area near central Lusaka. Chawama PHC is the only government-supported clinic in this community and is the primary source of medical care for Chawama residents, allowing for maximal study reach. NP swabs were obtained from infants at enrollment and approximately every two to three weeks thereafter through 14 weeks of age for a total of seven scheduled time points each. Mothers’ samples were gathered at all time points at which the infants were swabbed, but only the first and last time points at weeks zero to two and 12–14 were sequenced for analysis for a total of 40 samples.

**Table 1.** Baseline demographic characteristics of the full infant cohort, stratified by HIV exposure status. IQR refers to the interquartile range, BCG refers to the Bacille Calmette-Guérin vaccine for tuberculosis disease, and OPV refers to oral polio vaccine.

Parameter	HIV-Exposed	HIV-Unexposed	All Subjects
Number of Infants	10	10	20
**Place of Birth**			
University Teaching Hospital % (n)	40.0% (4/10)	30.0% (3/10)	35.0% (7/20)
Chawama Clinic % (n)	60.0% (6/10)	60.0% (6/10)	60.0% (12/20)
Chilenje Clinic % (n)	0.0% (0/10)	0.0% (0/10)	0.0% (0/20)
Home Delivery % (n)	0.0% (0/10)	10.0% (1/10)	5.0% (1/20)
Other % (n)	0.0% (0/10)	0.0% (0/10)	0.0% (0/20)
Male Sex % (n)	50.0% (5/10)	40.0% (4/10)	45.0% (9/20)
Median Age in Days at Enrollment (IQR)	6.0 (6–7)	6.5 (6–7)	6.0 (6–7)
**Gestational Age**			
Median Estimated Gestational Age at Delivery (weeks) (IQR)	40.0 (38–40)	40.0 (39–40)	40.0 (39–40)
Median Birth Weight (IQR)	3050.0 (2800–3200)	3100.0 (3000–3100)	3100.0 (2900–3200)
Twin Birth % (n)	0.0% (0/10)	0.0% (0/10)	0.0% (0/20)
**Immunizations at birth**			
BCG % (n)	30.0% (3/10)	50.0% (5/10)	40.0% (8/20)
OPV % (n)	30.0% (3/10)	20.0% (2/10)	25.0% (5/20)
Number of Mothers	10	10	20
Median Age in Years at Enrollment (IQR)	34.0 (29–36)	34.0 (30–37)	34.0 (30–37)
Married % (n)	100.0% (10/10)	100.0% (10/10)	100.0% (20/20)
Father Lives with Child % (n)	. % (./0)	. % (./0)	. % (./0)
**Labor and Delivery Complications**			
Obstructed Labor % (n)	0.0% (0/10)	0.0% (0/10)	0.0% (0/20)
Birth Asphyxia % (n)	10.0% (1/10)	0.0% (0/10)	5.0% (1/20)
Sepsis % (n)	0.0% (0/10)	0.0% (0/10)	0.0% (0/20)
Haemorrhage % (n)	0.0% (0/10)	0.0% (0/10)	0.0% (0/20)
Other Birth Complication % (n)	0.0% (0/10)	0.0% (0/10)	0.0% (0/20)
**Maternal Immunization**			
Median Number of Tetanus Toxoid Doses (IQR)	5.0 (3–5)	5.0 (3–5)	5.0 (3–5)
Received other vaccines % (n)	0.0% (0/10)	0.0% (0/10)	0.0% (0/20)
Received Influenza % (n)	0.0% (0/10)	0.0% (0/10)	0.0% (0/20)
Received TDaP % (n)	0.0% (0/10)	0.0% (0/10)	0.0% (0/20)
**Maternal HIV Status**			
Mother HIV(+) % (n)	100.0% (10/10)	0.0% (0/10)	50.0% (10/20)
Mother on ART % (n)	100.0% (10/10)	. % (./0)	100.0% (10/10)
Mother on ART Prior to Pregnancy % (n)	100.0% (10/10)	. % (./0)	100.0% (10/10)
**Trimester initiated ART**			
First Trimester % (n)	. % (./0)	. % (./0)	. % (./0)
Second Trimester % (n)	. % (./0)	. % (./0)	. % (./0)
Third Trimester % (n)	. % (./0)	. % (./0)	. % (./0)
**Household composition**			
Median Household Size (IQR)	5.5 (4–7)	6.0 (5–7)	6.0 (5–7)

HIV(+) mothers enrolled in the SAMIPS study were required to be on antiretroviral therapy (ART) to prevent mother-child transmission. Among the mothers in our immediate cohort, 50% (10/20) were HIV infected, of whom 100% had initiated antiretroviral treatment prior to conception. The study did not collect CD4 counts from study subjects, and assessment of mother’s HIV status relied upon previous testing done at the clinic. Although data on maternal HIV status was available, final HIV status could not be ascertained on the infants themselves, which typically is not possible until the infant is four to six months of age. As all mothers received ART during pregnancy, pooled transmission rates of breastfeeding mothers would be about 3.54% (95% CI: 1.15–5.93%) at the six month mark. ^19^ Therefore, it can be assumed that infants in this study becoming HIV(+) would be rare, and so all infants born to an HIV(+) mother were classified here as being HIV-exposed with two unknown subpopulations of HEU and HIV(+) infants. All enrolled infants received the pentavalent and pneumococcal vaccines at ages six, 10 and 14 weeks, which offer protection against 10 pneumococcal serotypes and *Haemophilus influenzae* type B. Additional information about the study structure and sampling methods can be found in Gill *et al*. (2016). ^18^

The HEU infants were restricted to subjects who had entered the study in April, May, June, or July and were matched with HUU infants on month of enrollment into study, mother’s age, and mother’s education. Some missingness in the data is present; for ten samples, these visits either never occurred or swabs were not collected. An additional three samples were excluded from analysis because fewer than 10,000 reads aligned to RefSeq reference genomes. This left us with 129 infant samples and 38 mother samples for analysis. In total, 16/20 infants had data for all seven time points, 2/20 infants had only six time points, one infant had three time points, and another infant had only two time points. All infants had swabs for the first and second time points. Only two of 20 mothers lacked both time points. The numbers of infant samples and mother samples collected for each time point are enumerated fully in Table 2.

**Table 2.** Counts and percentages of samples available for analysis from both infants and mothers. These are stratified by time point, infant age in weeks, and HIV status. HEU: HIV-exposed, uninfected; HUU: HIV-unexposed, uninfected.

	Infant	Mother	Overall
	( *n*=129)	( *n*=38)	( *n*=167)
**Average infant age (weeks)**			
0.58 (t=0)	20 (15.5%)	20 (52.6%)	40 (24.0%)
2.88 (t=1)	20 (15.5%)	0 (0%)	20 (12.0%)
6 (t=2)	19 (14.7%)	0 (0%)	19 (11.4%)
8.21 (t=3)	17 (13.2%)	0 (0%)	17 (10.2%)
10.21 (t=4)	17 (13.2%)	0 (0%)	17 (10.2%)
12.22 (t=5)	18 (14.0%)	0 (0%)	18 (10.8%)
14.35 (t=6)	18 (14.0%)	18 (47.4%)	36 (21.6%)
**HIV Status**			
HUU/HIV(-)	68 (52.7%)	19 (50.0%)	87 (52.1%)
HEU/HIV(+)	61 (47.3%)	19 (50.0%)	80 (47.9%)

### Sample processing and storage

NP swabs were obtained from the posterior nasopharynx using a sterile flocked tipped nylon swab (Copan Diagnostics, Merrieta, California). The swabs were then placed in universal transport media, put on ice and transferred to our onsite lab on the same campus, where they were aliquoted and stored at -80°C until DNA extraction. DNA was extracted using the NucliSENS EasyMagG System (bioMérieux, Marcy l’Etoile, France). Extracted DNA was stored at our lab located at the University Teaching Hospital in Lusaka at -80°C. Sample collection, processing and storage were previously described (Gill *et al*., 2016). ^18^

### 16S ribosomal DNA amplification and MiSeq sequencing

For 16S library preparations, two PCR reactions were completed on the template DNA. Initially the DNA was amplified using universal bacterial primers ^20^ specific to the V3–V4 region of the 16S rRNA gene. ^21^ Library preparation was performed according to the standard instructions of the 16S Metagenomic Sequencing Library Preparation protocol (Illumina, USA). The 16S primer pairs incorporated the Illumina overhang adaptor (16S forward primer 5’-TCGTCGGCAGCGTCAGATGTGTATAAGAGACAGCCTACGGGNGGCWGCAG-3’; 16S reverse primer 5’-GTCTCGTGGGCTCGGAGATGTGTATAAGAGACAGGACTACHVGGGTATCTAATCC-3’)

Each PCR reaction contained DNA template (~12 ng), 5µℓ forward primer (1μM), 5 µℓ reverse primer (1μM), 12.5 µℓ 2 X Kapa HiFi Hotstart ready mix (KAPA Biosystems Woburn, MA), and PCR grade water to a final volume of 25µℓ. PCR amplification was carried out as follows: heated lid 110°C, 95°C for 3 min, 25 cycles of 95°C for 30s, 55°C for 30s, 72°C for 30s, then 72°C for 5 min and held at 4°C. Negative control reactions without any template DNA were carried out simultaneously.

The size of the amplicons was then visualized using the 4200 TapeStation (Agilent Technologies, Germany). Successful PCR products were cleaned using AMPure XP magnetic bead-based purification (Beckman Coulter, IN). The IDT for Illumina Nextera DNA UD Indexes kit (Illumina, San Diego, CA) with unique dual index adapters were used to allow for multiplexing. Each PCR reaction contained purified DNA (5 μℓ), 10 μℓ index primer mix, 25 μℓ 2X Kapa HiFi Hot Start Ready mix and 10 μℓ PCR grade water. PCR reactions were performed on a Bio-Rad C1000 Thermal Cycler (Bio-Rad, Hercules, CA) Cycling conditions consisted of one cycle of 95°C for 3 min, followed by eight cycles of 95°C for 30 s, 55°C for 30 s and 72°C for 30 s, followed by a final extension cycle of 72°C for 5 min. PCR products of negative controls were confirmed negative on Agilent TapeSataion (no band observed).

Prior to library pooling, the indexed libraries were purified with Ampure XP beads and quantified using the Qubit dsDNA HS Assay Kit (Thermo Fisher Scientific, Waltham, MA). Purified amplicons were run on the Agilent TapeStation (Agilent Technologies, Germany) for quality analysis before sequencing. The sample pool (2 nM) was denatured with 0.2N NaOH, then diluted to 4 pM and combined with 10% (v/v) denatured 20 pM PhiX, prepared following Illumina guidelines. Libraries were then sequenced on the Illumina MiSeq sequencing platform (Illumina, USA) at the Sequencing Core Facility, National Institute for Communicable Diseases (NICD) of the National Health Laboratory Service, South Africa, using a 2 x 300 cycle V3 kit, following standard Illumina sequencing protocols. Negative controls were sequenced as well, resulting in extremely low reads that were not further analyzed.

In addition to using negative controls, all samples were processed at random to account for reagent contamination. Lab technicians were blinded to the timing of sample collection and clinical data.

We assessed the quality of the sequencing data using FastQC v0.11.9. ^22^ Trimmomatic ^20^ v0.39 was used to trim Illumina adapters and remove low-quality sequences. We performed a sliding window trim, cutting once when the average quality score within a window of six bases falls below 15. We removed both leading and trailing low quality or N bases below quality six. All other parameters used the default settings.

Sequencing data were aligned to bacterial genomes and profiled using the PathoScope 2.0 pipeline. ^23^ All RefSeq representative bacterial genomes available as of November 2, 2018 were used as a PathoScope reference library. We obtained target read counts delineated by National Center for Biotechnology Information (NCBI) unique identifiers (UIDs) and matched them to the NCBI Taxonomy database to retrieve accurate taxonomic hierarchy information. We then aggregated reads by genera. Data were transformed to relative abundances using counts per million (CPM) and normalized using log CPM for subsequent analyses. In most cases, taxa belonging to genera with average relative abundances of less than 1% were grouped as “Other” in analyses. All code has been made available via Zenodo. ^24^

### Statistical analysis

All analyses were performed using R Statistical Software (v4.2.1; R Core Team 2022).

Microbial abundances across sample groups were visualized using alluvial diagrams and stacked bar plots using the R packages ggplot2 v3.3.5 and alluvial v0.2-0 . The alluvial diagrams illustrate individual genera as stream fields that change position at different time points. The height of a stream field represents the relative abundance of that taxon. At a given time point, stream fields are ranked from the highest to lowest abundance (top to bottom). These were plotted for infants according to HIV exposure status over several time points. Stacked bar plots were used to visualize the relative abundance of microbes at a given taxonomic level in each sample, represented as a single bar, labeled by time point, and plotted within each HIV exposure status group for separate mothers and infant comparisons. These plotting and diagramming techniques allow for an efficient overview of the types of differences inherently present in the data at the group level.

Generalized estimating equations (GEEs) as described in Liang and Zeger (1986) ^25^ and extended by Agresti (2002) ^26^ have been widely used for modeling longitudinal data, ^27^ and more recently for longitudinal microbiome data. ^28, 29^ For each genus present in the microbial aggregate of samples, we modeled normalized log CPM relative taxon counts, estimating the effects of time point and HIV exposure status and their interaction, while accounting for the underlying structure of clusters formed by individual subjects. We defined the responses *Y*_1_, *Y*_2_,..., *Y _n_* as the collection of infant relative abundances for a given taxon in log CPM, with *n* = 129. We identified the mean model *µ _ij_* for the *i ^th^* infant and *j ^th^* timepoint. With regression parameters *β _k_* representing HIV exposure status and time point, and the AR(1) variance structure *V _i_*, we formed the estimating equation:

$$U(\beta )=\sum_{i=1}^{n}\frac{\partial \mu_{i}}{\partial \beta }\;V_{i}^{-1}\{Y_{i}-\mu_{i}(\beta )\}.$$

This then becomes an optimization problem, such that solving *U*( *β*) = 0 estimates the parameters *β _k_*. We modeled abundances for all 12 genera and nine of the top species. The link function *g* was chosen to be a Gaussian link. It was assumed that these abundances are correlated within infants for the various sampling time points. As such, we accounted for this with a first-order autoregressive AR(1) working correlation structure with homogenous variances such that the correlation between adjacent time points was assumed to be more similar. Parameter estimates were collected from each model, along with Wald test p-values. Multiple testing was adjusted for using a Bonferroni correction. Models were created in R using geepack v1.3.10. ^30^

Hotelling’s *T* ^2^ tests ^31^ were used to determine whether the microbiome profiles exhibited notable differences or trends across time and groups. We then used t-tests to identify which genera contributed most to these differences. Groups of mothers and infants or HEU and HUU infants are designated as the two sampling units on which the relative abundances of the *p* most abundant genera were measured. For paired tests, we chose *p* = 6 variables to ensure that *n* < *p* so that singularity could be avoided and *T* ^2^ could be properly computed, where *n* is the number of measurements in a sampling unit. We generally tested the hypotheses

$$H_{0}:\mu_{y}=\mu_{x}\;\text{vs.}\;H_{A}:\mu_{y}\neq \mu_{x}$$

which, in the paired case given *μ _diff_* = *μ _y_* – *μ _x_*, is equivalent to

$$H_{0}:\mu_{diff}=0\;\text{vs.}\;H_{A}:\mu_{diff}\neq 0$$

to conduct a comparison between groups for the *p* most abundant genera for testing groups. The population means *μ _x_* and *μ _y_* represent the two sampling units of a given test, which were generally either *μ _mothers_* and *μ _infants_* or *μ _HUU_* and *μ _HEU_*. We treated samples as paired in both cases, such that the mothers are paired with their own infants, or HEU and HUU infants were paired according to the pre-analysis matching carried out previously. When conducting testing on data from HIV positive mothers and HIV negative mothers, we did not pair mothers and instead relied on a standard two-sample test. We assumed that the relevant conditions for testing are met, meaning that the groups are correlated and have a multivariate normal distribution. Normality is met by using microbe abundances in log CPM units.

We conducted three main groups of testing with the *T* ^2^ statistic. The first paired test was between mother-infant pairings at the first or last time point ( *t* ∈ [0, 6]) and for either the mother/infant HIV(+)/HEU or HIV(-)/HUU groups for a total of four tests. These tests offer a clear between-group comparison while accounting for similarity in the mother/infant dyads and calculating genera-specific differences in ruling whether the hypotheses are met. The second testing approach consisted of seven tests. We compared relative abundances of the six most highly abundant genera at each of the seven time points ( *t* ∈ [0, 1,..., 6]) from paired HEU and HUU infants. The final testing approach compared all HIV(+) and HIV(-) mothers’ samples for all 12 genera across mothers, including the “Other” designation as described previously. All samples were unpaired and therefore utilized the unpaired multivariate *T* ^2^ generalization.

Test statistics were calculated to test whether microbiome profiles in paired infants were notably different among the seven time points, and whether HEU mothers and infants had similar profiles at the first and last time points. After calculating the *T* ^2^ test statistic, we calculate an equivalent *F _p,n–p_* test statistic distributed with *p* and *n* – *p* degrees of freedom and use this distribution to calculate the test’s p-value:

$$\text{p-value}=\text{Pr}\left\{F_{p,n-p+1}>\frac{n-p+1}{np}T^{2}\right\}$$

Paired or two-sample t-tests were used to distinguish which genera are most important to the differences identified with Hotelling’s *T* ^2^ tests, and *α*-levels were adjusted after performing the *p* tests by using a Bonferroni critical value.

Beta diversity using the Bray-Curtis dissimilarity metric was compared using a non-parametric Wilcoxon rank-sum test between HEU and HUU infants, and between HIV(+) and HIV(-) mothers. Tests were conducted separately for each subset of samples for the first and last time points for a total of four tests. The null hypothesis conjectures that the distributions of both populations are equal, under the general assumption that all observations from both groups are independent of each other.

## Results

### Sample characteristics

Counts of infant and mother samples available at each time point and for each group of interest can be found in Table 2. Although we included 10 HUU and 10 HEU infants and their mothers in the study, there was some small variation in numbers of samples at the different time points, but they were overall close in evenness. Infants were approximately 7 ± 1 (mean ± SD) days old at time of first swabbing, and were on average 22 ± 2 days, 42 ± 3 days, 58 ± 2 days, 74 ± 2 days, 88 ± 2 days, and 105 ± 2 days respectively at subsequent time points 1–6.

### Longitudinal differences in the nasopharyngeal microbiome composition

**Figure 1.**
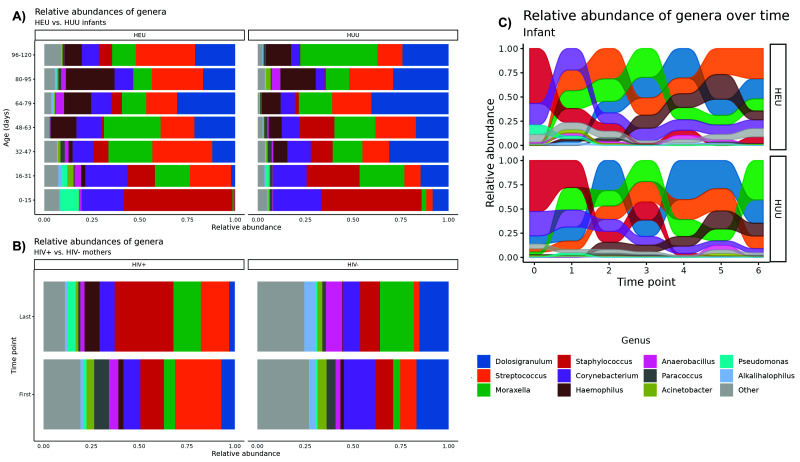
The maturation over 120 days of the NP microbiomes of **A**) healthy HIV-exposed, uninfected (HEU; n=10) and HIV-unexposed, uninfected (HUU; n=10) infants and **B**) HIV(+) (n=10) and HIV(-) mothers (n=10). These stacked bar plots reveal variation in the relative abundance of microbes between groups of either infants or mothers clustered at the genus level. Each bar represents a single time point binned by age and is the average of ~10 samples. Genera with an average relative abundance of <1% across all samples are labeled as “Other.” The alluvial plot in **C**) depicts the changing presence of genera across all infant samples by HIV exposure status. As relative abundances change over time, the position of a flow stream representing a single genus may change position relative to the other genera.

Our FastQC analysis indicated that the overall sequencing quality was excellent, with mean Phred quality scores remaining greater than 25 (99.5% accuracy) for at least 175 bp for both forward and reverse reads. Trimmomatic removed less than 3% of reads in any given sample. The analysis covered 129 infant and 38 mother swab samples, with an average of 124,905 ± 299,120 (mean ± SE) reads per infant sample (max = 3,016,276; min = 13,218) and 70,463 ± 48,730 reads per mother sample (max = 208,029; min = 14,339). The number of reads in infants’ samples was significantly higher than that of mothers’ swabs (p = 0.02), which may in part be the result of mothers’ acquired immunity over time and therefore lower overall NP carriage. In our raw data, we uniquely identified 17 phyla, 647 genera, and 758 total species across all samples. NP microbiome composition separated according to HIV exposure status are depicted in Figures 1A and 1B for infants and mothers, respectively. The alluvial plot in Figure 1C graphically depicts the most abundant genera in log CPM present in HEU and HUU infants. As relative abundances (in log CPM) change over time, the position of a flow stream representing a single genus may change position relative to the other genera. At a given time point, the flow streams are stacked according to the abundance relative to other streams.

Due to the heavy presence of taxa with relatively low abundance, we labeled taxa with average relative abundances of less than 1% as “Other” at the genera and species level. Post-grouping, the present taxa were limited to three distinctly identifiable phyla, encompassing 12 genera and 87 species. The most abundant phyla were the Firmicutes (~56.5%), Proteobacteria (~22.4%), and Actinobacteria (~16.1%). The remaining 5% of reads were characterized as “Other.” At the genus level, the most abundant groups were the *Dolosigranulum* (~23.5%), *Staphylococcus* (17.2%), *Corynebacterium* (~16.1%), *Streptococcus* (~13.5%) and *Moraxella* (~12.4%) genera.

For both the HEU and HUU infant groups, the respiratory microbiome during the first months of life is dominated by *Staphylococci* and *Corynebacteria.* Early on, we observed the emergence of more typical respiratory bacteria such as *Moraxella* and *Streptococcus* sp. Additionally, the commensal *Dolosigranulum* emerges as a dominant member of the microbiome within the first weeks of life. We saw the appearance of *Haemophilus* somewhat later at around four to six weeks. Overall, these transitions occurred in an orderly and stepwise pattern. These transitional patterns align with those found in a longitudinal East Asian infant cohort. ^32^

While longitudinal trends were strongly apparent across infant groups, the observed differences between the HEU and HUU infants were subtle. Figure 1A illustrates higher amounts of *Streptococcus* and suppression of *Dolosigranulum* among HEU vs. HUU infants. Furthermore, Figure 1C suggests increasing amounts of *Streptococcus* and *Haemophilus* over time for HEU infants, whereas *Staphylococcus* largely declines after time point three in both groups. Together these findings support a time-dependent effect on genus abundance taking place soon after birth.

As this is one of the few studies conducted on the NP microbiome in Zambia, there are no conclusive baseline expectations for a typical microbiome composition. However, we have compiled the results of a published longitudinal study that analyzed NP swabs from a cohort of 234 healthy infants from Washington, D.C. ^33^ ( Table 3). Swabs in the study were taken at ~two, ~six, and ~12 months. We have included the proportion of genera present across the aggregate of all swabs, and for the ~two-month sampling (no further information on exact timing of swabs was specified). Slight differences appear to be present, which may be in part due to differences in living conditions, sample size, and choice of analysis database used. The Teo *et al*. study utilized the Greengenes database, which produces far less sensitive results when compared to other databases like RefSeq and Silva. ^34^ Otherwise, when comparing the Teo *et al*. cohort at two months to those in our study, we find that the abundance of *Staphylococcus* is nearly twice as high as in our study for either HEU or HUU infants, although the *Corynebacterium*, *Moraxella*, *Streptococcus* average means are all within a few percentage points for both groups. In our analysis, the most overwhelming difference between the HEU and HUU infants is in *Dolosigranulum*, for which HUU infants showed a 19.63% higher presence of *Dolosigranulum* when averaged across time points. Overall, as this study is not a one-to-one comparison to the Teo *et al*. study, it is difficult to draw exact conclusions, but it appears that the NP microbiome profiles observed here are similar to those seen in healthy infants of a similar age.

**Table 3.** Comparison of average genera relative abundance with a NP longitudinal study of a healthy infant cohort from Washington, D.C. The number of samples, age group by month (m), sequenced 16S region, patient condition and genus mean relative proportions are enumerated. While the studies are not a one-to-one comparison, the relative abundances between studies appear to be similar. NR denotes statistic not reported in original paper.

Study	Teo *et al*. (2015) ^33^	Teo *et al*. (2015) ^33^	This study	This study
Number of samples	1,021	~177	68	61
Age group	2–12 m	2 m only	0–3.5 m	0–3.5 m
16S Region	V4	V4	V3–V4	V3–V4
Patient condition	Healthy	Healthy	Healthy	HEU
**Microbe**				
*Dolosigranulum*	8.80%	14%	32.04%	12.41%
*Streptococcus*	14%	14%	8.93%	14.54%
*Moraxella*	31.20%	9%	10.65%	15.65%
*Staphylococcus*	10.30%	41%	19.71%	14.54%
*Corynebacterium*	13.50%	22%	17.92%	14.93%
*Haemophilus*	9.70%	NR	4.91%	9.21%
*Anaerobacillus*	NR	NR	0.94%	2.02%
*Paracoccus*	NR	NR	1.20%	0.57%
*Acinetobacter*	13%	NR	1.10%	0.70%
*Pseudomonas*	NR	NR	0.29%	3.55%
*Alkahilophilus*	NR	NR	0.15%	0.67%
Other	NR	NR	2.16%	5.64%

### Relationship between nasopharyngeal microbiome composition and HIV exposure

**Figure 2.**
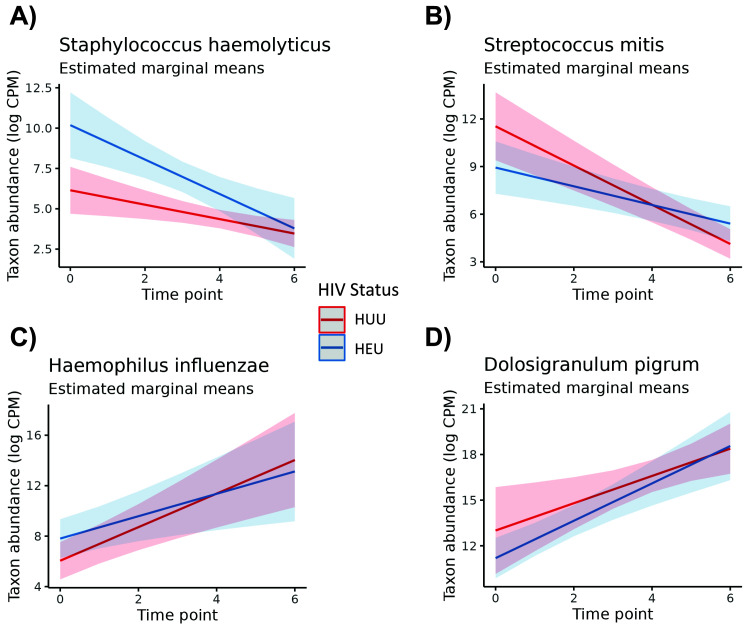
Plots of estimated marginal means of the abundance of a given microbe accounting for estimated GEE effects. The time of sampling is on the x-axis, and the abundance of the microbe in log CPM is on the y-axis. The estimated change in abundance over time is illustrated for control infants (in red) and HIV-exposed, uninfected (HEU) infants (in blue). The interaction between time and HIV exposure status is accounted for in the differing group slopes. All microbes had at least marginally significant time effects, but only *S. haemolyticus* had a very strong HIV exposure status effect.

GEEs revealed some differences among genera and species for time point and HIV exposure status when adjusting for subject variation and the interaction effect. Models were created for all 12 genera (including the “Other” grouping) and for the nine species that averaged greater than 1% abundance across all infant samples. Of all taxa tested, four of nine species and six of 12 genera exhibited substantial instability across time points ( Table 4). One species, *Staphylococcus haemolyticus*, was highly associated with HIV exposure in infants (p <0.01, adj. p = 0.01). Additionally, *Streptococcus mitis* indicated a strong interaction effect between time and HIV exposure status (p <0.01, adj. p = 0.04). Figure 2 shows the estimated marginal means of the time and HIV-exposure status effects for *S. haemolyticus*, *S. mitis*, *Haemophilus influenzae*, and the *Dolosigranulum* genus. We chose to include these microbes in the figure as a representative selection of microbes with significant and non-significant effects. In Figure 2a, differences in the abundance of *S. haemolyticus* for HEU and HUU infants is cleanly pronounced for several time points, reflecting the Wald test result, but this is not the case for the other microbes in Figures 2b, 2c, or 2d. All of these had at least marginally significant p-values when testing the time point effect but lacked strong HIV exposure status effects.

**Table 4.** Table of P-values from Wald tests on GEE models. The effects modeled were HIV status, and time point effects, with their interaction. Results are stratified by species and genus. All p-values less than alpha=0.05 are bolded.

	Time point Unadjusted p-values	Time point Coefficient Estimate	Time point Adjusted p-values	HIV Status Unadjusted p-values	HIV Status Coefficient Estimate	HIV Status Adjusted p-values	Interaction Unadjusted p-values	Interaction Coefficient Estimate	Interaction Adjusted p-values
**Genus**									
*Acinetobacter*	**0.00**	-0.09	**0.02**	0.78	-0.06	1.00	0.29	0.04	1.00
*Alkalihalophilus*	**0.01**	0.18	0.07	0.15	0.67	1.00	**0.03**	-0.26	0.30
*Anaerobacillus*	0.19	-0.11	1.00	0.12	0.77	1.00	0.56	-0.06	1.00
*Corynebacterium*	**0.01**	-0.18	0.09	0.11	-0.39	1.00	0.18	0.12	1.00
*Dolosigranulum*	**0.01**	0.25	0.13	0.35	-0.46	1.00	0.46	0.09	1.00
*Haemophilus*	**0.00**	0.43	**0.00**	0.14	0.69	1.00	0.23	-0.18	1.00
*Moraxella*	**0.00**	0.35	**0.00**	0.21	0.54	1.00	0.07	-0.14	0.89
*Other*	**0.03**	-0.09	0.30	0.81	-0.04	1.00	0.25	0.06	1.00
*Paracoccus*	0.44	-0.04	1.00	0.08	-0.50	0.99	0.09	0.12	1.00
*Pseudomonas*	**0.00**	-0.15	**0.00**	0.19	-0.52	1.00	**0.03**	0.17	0.40
*Staphylococcus*	**0.00**	-0.38	**0.00**	0.53	-0.13	1.00	0.35	0.04	1.00
*Streptococcus*	**0.00**	0.13	**0.01**	0.43	-0.18	1.00	0.12	0.09	1.00
**Species**									
*Corynebacterium accolens*	0.52	-0.27	1.00	0.26	-2.34	1.00	0.18	0.70	1.00
*Corynebacterium* * pseudodiphtheriticum*	0.58	0.25	1.00	0.30	-1.91	1.00	0.34	0.51	1.00
*Dolosigranulum pigrum*	**0.01**	0.90	0.06	0.26	-1.82	1.00	0.42	0.33	1.00
*Haemophilus influenzae*	**0.00**	1.33	**0.00**	0.11	1.76	0.96	0.30	-0.44	1.00
*Staphylococcus epidermidis*	**0.03**	-0.57	0.24	0.97	-0.07	1.00	0.56	-0.21	1.00
*Staphylococcus haemolyticus*	**0.01**	-0.45	0.07	**0.00**	4.04	**0.01**	0.07	-0.62	0.59
*Staphylococcus simiae*	**0.00**	-1.27	**0.01**	0.11	-4.49	1.00	0.08	0.88	0.69
*Streptococcus mitis*	**0.00**	-1.24	**0.00**	0.06	-2.60	0.53	**0.00**	0.65	**0.04**
*Streptococcus pneumoniae*	**0.00**	1.81	**0.00**	0.16	1.85	1.00	0.52	-0.20	1.00

Hotelling’s *T* ^2^ test statistics were used to compare relative abundances of the top six most abundant genera between paired HEU and HUU infants at each time point, namely *Dolosigranulum*, *Streptococcus*, *Moraxella*, *Staphylococcus*, *Corynebacterium*, and *Haemophilus*. As previously noted, we chose *p* = 6 variables to ensure that *n* < *p* so that singularity could be avoided and *T* ^2^ could be properly computed, where *n* is the number of measurements in a given sampling unit. Paired infants did not present significantly different microbiome profiles at any of the time points at *α* = 0.05 ( Table 5). The largest differences seemed to occur at time point 5 (p = 0.07). The fluctuating critical *F* values indicate that uneven sample distribution across time points contributes to the difficulty in identifying clear differences between populations.

**Table 5.** Table of Hotelling’s T ^2^ test results. Tests compared log CPM relative genera abundances of paired HIV-exposed, uninfected (HEU) and HIV-unexposed, uninfected (HUU) infants at all seven time points. The first and second degrees of freedom for the test are denoted by *df*_1_ and *df*_2_, respectively.

	Time point 0	Time point 1	Time point 2	Time point 3	Time point 4	Time point 5	Time point 6
*df*_1_	6	6	6	6	6	6	6
*df*_2_	4	4	3	1	1	2	2
**Critical *F* value**	6.16	6.16	8.94	233.99	233.99	19.33	19.33
***F* statistic**	0.90	1.99	0.47	3.41	22.66	14.56	1.26
**p-value**	0.57	0.26	0.80	0.39	0.16	0.07	0.50

Overall microbiome composition varied significantly at the times of first and last sampling for HUU and HEU infants. Among all infant samples at time point 0, a Wilcoxon rank-sum test of the Bray-Curtis dissimilarity revealed a non-significant difference in microbiome composition between HUU and HEU infants (p = 0.68). The same test for all infant samples at time point six was contrastingly showed large differences in beta diversity (p <0.01). Within-group, between-subject tests revealed variation at the first and last time points within the HEU group, and at both first and last time points within the HUU group (all p-values < 0.01). However, large variation was not present between time points within the HEU (p = 0.11) or HUU infant samples (p = 0.78). As a result of heavy inter-subject variability, alpha diversity metrics were not deemed useful for this analysis.

### Relationship between infant and mother nasopharyngeal microbiome composition

We also investigated the relationship between the NP microbiome in infants and their mothers. Paired Hotelling’s *T* ^2^ tests were again used to test the log CPM of genera as a measure of relative abundance at the first and last time points for the HIV(+)/HEU and HIV(-)/HUU groups ( Table 6). Genera tested were the same as listed for the infant-specific Hotelling’s tests, delineated previously. HIV(+)/HEU mother-infant pairs had marginally significant profile differences at the first time point ( $T_{6,4}^{2};$ p = 0.10) but were similar enough to not present as having significant profiles at the last time point ( $T_{6,1}^{2};$ ; p = 0.60). HIV(-)/HUU mother-infant pairs also had marginally significant differences at the first time point ( $T_{6,4}^{2};$ p = 0.10) but varied notably at the last time point ( $T_{6,3}^{2};$ p = 0.02). Paired t-tests for the six genera were separately conducted for HIV(-)/HUU mother-infant pairs to distinguish which genera are most important to the identified difference at that time point. The largest difference in abundance occurred for the *Haemophilus* ( *t*_8_, p = 0.02; adj. p-value = 0.10) and *Staphylococcus* genera ( *t*_9_; p-value <0.01; adj. p-value <0.01). HUU Infants were noted as having greater *Haemophilus* carriage than their HIV(-) mothers, whereas mothers had greater *Staphylococcus* carriage than their infants.

**Table 6.** Table of Hotelling’s T ^2^ test results. Tests compared log CPM relative genera abundances at the first and last time points for the HIV(+)/HEU and HIV(-)/HUU groups using mother-infant pairs. HEU: HIV-exposed, uninfected; HUU: HIV-unexposed, uninfected.

	Time point 0		Time point 1	
	HEU/HIV(+)	HUU/HIV(-)	HEU/HIV(+)	HUU/HIV(-)
*df*_1_	6	6	6	6
*df*_2_	4	4	1	3
**Critical *F* value**	6.16	6.16	233.99	8.94
***F* Statistic**	4.13	4.14	1.20	19.52
**p-value**	0.10	0.10	0.60	**0.02**

As part of our inquiry into why HEU infants might be more susceptible to higher morbidity or mortality, we conducted paired t-tests for well-known pathogenic species between mother-infant pairs for *Streptococcus pneumoniae*, *Haemophilus influenzae*, and *Staphylococcus haemolyticus*. Tests compared pairs at the first and last time points, and within either HIV or control subgroups, for a total of four different tests per species ( Table 7). Inequality in pathogen carriage was ascertained by the mean of differences in log CPM. The tests for *S. pneumoniae* were nominally significant at *t* = 0 for the HIV(+)/HEU group (p = 0.03, adj. p = 0.4) and *t* = 6 for the HIV(-)/HUU group (p = 0.01, adj. p = 0.1). In the former, the mothers had more pathogen carriage than the infants, but this was the opposite case for the latter. *H. influenzae* was more highly abundant in HUU infants than in their HIV(-) mothers at *t = 6* (p = 0.01, adj. p = 0.1), and *S. haemolyticus* was likely to be found in the HUU infants at t = 0 in a higher concentration than in their mothers.

**Table 7.** Results table from paired t-tests of log CPM relative abundances of well-known pathogenic species. Tests compared mother-infant pairs at the first and last time points, and within either HIV or control subgroups, for a total of four different tests per species. Lower and upper 95% confidence interval bounds are denoted by the Lower CI and Upper CI columns. The test degrees of freedom are denoted by *df*.

Species	HIV Status	Time point	p-value	Lower CI	Upper CI	Mean of differences (log CPM)	*t*	*df*	Adjusted p-value
*Streptococcus pneumoniae*	HIV	0	**0.03**	0.26	3.80	2.03	2.60	9	0.4
		6	0.16	-3.47	0.73	-1.37	-1.60	6	1.0
	Control	0	0.12	-0.43	3.28	1.42	1.73	9	1.0
		6	**0.01**	-4.86	-0.79	-2.83	-3.20	8	0.1
*Haemophilus influenzae*	HIV	0	0.84	-1.55	1.87	0.16	0.21	9	1.0
		6	0.38	-3.87	1.70	-1.08	-0.95	6	1.0
	Control	0	0.88	-1.18	1.36	0.09	0.15	9	1.0
		6	**0.01**	-2.69	-0.45	-1.57	-3.23	8	0.1
*Staphylococcus haemolyticus*	HIV	0	0.03	-4.36	-0.27	-2.31	-2.56	9	0.4
		6	0.40	-0.94	2.06	0.56	0.91	6	1.0
	Control	0	**0.01**	-3.02	-0.61	-1.81	-3.41	9	0.1
		6	0.27	-0.52	1.60	0.54	1.17	8	1.0

### Trends in NP microbial community composition between HIV(+) and HIV(-) mothers

We compared HIV(+) and HIV(-) mothers at the time points that we sequenced. We used the Bray-Curtis dissimilarity metric to compare compositional dissimilarity between NP microbiomes. We identified strong dissimilarity between HIV(+) and HIV(-) mothers at time point six (p <0.01), but higher similarity at the first time point (p = 0.70). Additionally, the summed abundances of all twelve genera, including “Other” genera, were used to conduct a two-sample Hotelling’s *T* ^2^ test on unpaired mothers. The differences we observed were marginally significant at the first time point ( $T_{12,7}^{2};$ p = 0.16), but more noticeable at the latter ( $T_{12,5}^{2};$ p = 0.04). Unpaired *t*-tests indicated that the “Other” taxa (p = 0.03, adj. p = 0.37), *Alkalihalophilus* (p = 0.08, adj. p = 1), and *Paracoccus* (p = 0.10, adj. p=1) were the largest contributors to this difference. These taxa were all more highly abundant in the HIV(-) mothers.

## Discussion

To date, many studies have examined the effect of the microbiome of HIV(+) mothers on their infants and found noticeable differences. Bender *et al*. (2016) reported that although very few differences were apparent in the microbiomes of mothers with and without HIV infection, maternal HIV infection was associated with changes in the mouth, skin, and gut microbiome of HEU infants from Haiti. ^35^ Higher abundance of *Pseudomonadaceae* and *Thermaceae*, along with decreased bacterial diversity in stools of HEU infants was suggested as one mechanism that accounts for the immunologic derangements and poor growth observed in these children. It has been noted that the microbiome of mother and infant dyads reveals some associations with HIV infection, ^36^ particularly that infants’ microbiomes reflect the dysbiosis of their mothers, but how this dysbiosis is established in the HEU infant is poorly understood. Significantly higher bacterial diversities have been found in the fecal matter of HIV-exposed infants, compared to HIV-unexposed infants in an African cohort. ^37^ The relatively small number of studies looking specifically at the microbiome of the nasopharynx in HIV-exposed infants have found few changes associated with HIV infection, ^38^ even though increased risk of pneumococcal colonization and disease remains apparent for these infants. ^7, 13, 14^ Although dysbiosis seems to be a reasonable factor in this risk, the few available data contradict this hypothesis, demonstrating no differences in pathogen carriage between children with HIV infection and control groups. ^15– 17^

Our study explored differences in the NP microbiome among Zambian HEU and HUU infants and their mothers over a three-month period. Our starting point for this analysis was to better understand the observed excess mortality and increased rates of respiratory disease among HEU infants. Assuming that such differences are not merely due to sampling biases, the chief hypotheses explaining them are that 1) HEU infants have subtle immunological deficits; or 2) these are the consequence of confounding due to environmental and sociological factors. These are not mutually exclusive. We had reasoned that shifts in the microbiome between HEU and HUU infants would be harder to explain based on sociology and more likely reflective of the external environment and immunological factors.

Our analyses indicate that the HEU infants in our study exhibited subtle differences in the NP microbial composition throughout the sampling interval. Given our limited number of samples, it is within reason that these differences are a result of the sample variation. Although we cannot exclude true differences between the populations, we are left with uncertain evidence that there is an HIV exposure effect on the NP microbiome in the first 14 weeks after birth.

Initial plots of the microbial relative abundances appeared to show dynamic changes in abundances of certain genera; it is apparent from Figure 1A and 1C that *Staphylococcus* and *Corynebacterium* began as primary colonizers of the NP microbiome but were in time replaced by *Dolosigranulum*, *Streptococcus*, *Moraxella* and *Haemophilus*. The plots appear to show *Dolosigranulum* may have higher carriage with simultaneous lower carriage of *Streptoccocus* in HUU infants. From Figure 1B, we observe that HIV(+) mothers may have higher Streptococcus abundance than HIV(-) mothers. The decreased presence of “Other” genera indicates greater bacterial diversity in HIV(-) mothers’ NP microbiomes over HIV(+) mothers.

Some of these observations are supported by the findings from the GEE modeling ( Table 4). For instance, some taxa appeared to be present in larger proportions at certain time points for HEU or HUU infants, but in general, taxa seemed to follow the same general trends in both groups. Regardless of HIV exposure status, it was apparent that as the child grows, an increasing amount of respiratory flora emerges. As time went on, we found that HEU infants diverged in their microflora profiles and diversity from the HUU infants, as was shown by the Wilcoxon test at the last time point. Interestingly, this was not verifiable in the multivariate Hotelling’s T-squared test that collectively tested the top six of the most prevalent genera over time. This indicates that differences may have occurred for individual genera at a given time point yet did not result in holistic trends involving several of the top genera. The largest difference seemed to occur at time point five. It would seem reasonable to suggest that at birth, infants’ microbiome profiles are more similar than different, and that the diversification of these profiles occurs over time; however, the lack of any considerable difference at time point six disputes this argument.

When comparing HEU and HUU infants, the only microbe that appeared to have a distinct presence was that of *Staphylococcus haemolyticus*. The microbe indicated a distinctive high association with HIV exposure at birth, even when accounting for the interaction between HIV exposure status and time point. In the microbiome, *Staphylococcus haemolyticus* is not known for any pathogenic characteristics. Yet its relative taxonomic proximity to other pathogenic species of *Staphylococcus* suggest that a library misidentification, as is possible with any genomic reference library, ^34^ could result in identifying one or more other *Staphylococcus* species as *S. haemolyticus*. There is a possibility that our genomic identification process could be misidentifying what are actually *S. aureus* reads. *S. aureus* is one of the most common pathogens colonizing the nasopharynx and the lower airways, ^39, 40^ and carriage has been found to be significantly higher in RSV than rhinovirus-infected infants. ^41^ In a case series from India, NP carriage densities of *Streptococcus pneumoniae* and *S. aureus* were higher in both mothers and children living in HIV-affected households, regardless of the child's HIV status. ^42^

From the stacked barplots, we observed that *Dolosigranulum* seemed to have a higher relative proportion at all time points in HUU infants, but this was not verified as a distinguishable difference in our statistical testing. Yet, it would make sense for this trend to occur, as high *Dolosigranulum* carriage has been found to be correlated with postive outcomes of RSV, ^43^ COVID-19, ^44^ HIV exposure, ^45^ and Bronchiolitis. ^46^ It is generally accepted as a marker of a healthy microbiome, positively associated with *Corynebacterium* and potentially protective against colonization by *S. aureus* and *S. pneumoniae*. ^47^ Yet, it is unclear as to what *Dolosigranulum*’s exact role is in mediating defense against illness.

When comparing infants to their mothers with paired analyses, HEU infants’ NP microbiome composition was not vastly different from their HIV(+) mothers at birth or 14 weeks, including in their carriage of *S. pneumoniae*, *H. influenzae*, and *S. haemolyticus*. HUU infants were similar to their mothers at birth, but apparently grew apart from their HIV(-) mothers by 14 weeks as infants acquired more *Haemophilus* (specifically, *Haemophilus influenzae*) and decreased in *Staphylococcus* carriage.

As we had sequenced samples from both HIV(+) and HIV(-) women as part of this study, we were interested to see if we could ascertain any general differences from the present samples. We found that the two groups of women had greater dissimilarity at the last time point than the one immediately after birth, with HIV(-) women exhibiting a larger presence of taxa with relatively small abundances (denoted as “Other” in our analyses) revealing more microbial diversity in their NP microbiomes. In the nasopharynx, lower diversity has been associated with individuals with rhinovirus illness ^48^ and in children with HIV-associated bronchiectasis, ^49^ suggesting that greater NP diversity is a sign of a healthy microbiome, mirroring a previous finding in the gut. ^50^

Based on our results, we see some nuance, but must acknowledge that our study has several limitations. First, and most importantly, given this is both a pilot and a longitudinal study, our sample size is small. Second, this is a retrospective analysis of samples that were taken for the purpose of detecting *Bordetella pertussis* and not aimed for microbiome analysis. Nonetheless, the samples were collected in appropriate transport media and were transferred immediately on ice to the research lab. Our quality control analysis confirmed high quality of the sequencing data. Lastly, as the SAMIPS study was not focused on studying HIV transmission as a main aspect of its structure, several key variables were not measured that would have been informative in studying our results. For example, we do not know the viral loads and CD4 counts of the mothers at any point, and we lack information on whether mothers continued to take ART post-pregnancy. We are also unable to determine whether infants were infected with HIV as proper testing to determine transmission was not conducted as part of the study. On this last point, however, even if infants were HIV(+) rather than HEU, this sampling would have occurred at an early stage in disease and are unlikely to be highly immunologically deficient. Further, the direction of bias would magnify the differences between the two groups rather than diminish them, supporting the differences we saw in our analysis.

These limitations, and especially the small sample sizes, also negatively affect the power of our testing and modeling efforts. This is intended as a case study for learning about what trends may be present in this cohort that merit further research with more samples for comparison. It is also of interest as few microbiome studies are longitudinal, providing us with a new perspective on how the NP microbiome may be characterized across time in the different groups.

## Conclusions

Acknowledging these issues, our findings may suggest that that sociological differences between populations could mask more subtle inter-group nuances. This warrants further research and discussion regarding the HIV exposure phenomenon before readily affirming or denying that HIV exposure affects infants’ NP microbiomes. The potential effect of the HIV(+) mother’s microbiome on an infant may present further changes in pathogen carriage, or community diversity, as was suggested in our analysis. If associations hold, identifying HIV exposure as a predisposing factor to illness and poor health in infants would present opportunities for further research and development to support infants’ living situations, especially in areas of higher HIV transmission.

## Consent

Written informed consent for publication of the patients’ details was obtained from the parents of the patients.

## Data Availability

Zenodo: Underlying data for ‘Characterization of longitudinal nasopharyngeal microbiome patterns in maternally HIV-exposed Zambian infants’. https://doi.org/10.5281/zenodo.7255313 ^24^ This project contains the following underlying data: Data file 1: FinalDatOther.rds Data file 2: FinalDatPICRUSt2.RDS Data file 3: animalculesFinalHIV.rds Data file 4: animalcules_data_2021.rds Data file 5: mappingFaits.csv Data file 6: mappingFinalHIV.csv Data file 7: mappingFinalHIV.tsv Data file 8: preclean_MetaData.txt Data file 9: samips_immunization.csv Data files 10–176: *-sam-report.tsv Data are available under the terms of the Creative Commons Zero “No rights reserved” data waiver (CC0 1.0 Public domain dedication). NCBI BioProject: Characterization of longitudinal nasopharyngeal microbiome patterns in maternally HIV-exposed Zambian infants. Accession number PRJNA874826. https://identifiers.org/NCBI/BioProject:PRJNA874826

## Software availability

Source code available from: https://github.com/aubreyodom/HIV_Exposed_Infants

Archived source code at time of publication: https://doi.org/10.5281/zenodo.7255313 ^24^

License: CC0 1.0 Public domain dedication
