# Characterization of longitudinal nasopharyngeal microbiome patterns in maternally HIV-exposed Zambian infants

**DOI:** 10.12688/gatesopenres.14041.2

**Published:** 2024-08-30

**Authors:** Aubrey R. Odom, Christopher J. Gill, Rachel Pieciak, Arshad Ismail, Donald Thea, William B. MacLeod, W. Evan Johnson, Rotem Lapidot

**Affiliations:** 1 Bioinformatics Program, Boston University, Boston, MA, 02118, USA; 2 Department of Global Health, Boston University School of Public Health, Boston, MA, 02118, USA; 3 Sequencing Core Facility, National Institute for Communicable Diseases of the National Health Laboratory Service, 2131 Johannesburg, South Africa; 4 Department of Biochemistry and Microbiology, University of Venda, Thohoyandou 0950, South Africa; 5 Division of Infectious Disease, Center for Data Science, Rutgers New Jersey Medical School, Newark, NJ, 07103, USA; 6 Pediatric Infectious Diseases, Boston Medical Center, Boston, MA, 02118, USA; 7 Department of Pediatrics, Boston University School of Medicine, Boston, MA, 02118, USA

**Keywords:** nasopharyngeal microbiome, longitudinal cohort study, microbial communities, HIV exposure, children

## Abstract

**Background:**

Previous studies of infants born to HIV-positive mothers have linked HIV exposure to poor outcomes from gastrointestinal and respiratory illnesses, and to overall increased mortality rates. The mechanism behind this is unknown, but it is possible that differences in the nasopharyngeal (NP) microbiome between infants who are HIV-unexposed or HIV-exposed could play a role in perpetuating some outcomes.

**Methods:**

We conducted a longitudinal analysis of 170 NP swabs of healthy infants who are HIV-exposed (n=10) infants and their HIV(+) mothers, and infants who are HIV-unexposed, uninfected (HUU; n=10) .and their HIV(-) mothers. These swabs were identified from a sample library collected in Lusaka, Zambia between 2015 and 2016. Using 16S rRNA gene sequencing, we characterized the maturation of the microbiome over the first 14 weeks of life to determine what quantifiable differences exist between HIV-exposed and HUU infants, and what patterns are reflected in the mothers' NP microbiomes.

**Results:**

In both HIV-exposed and HUU infants, *Staphylococcus* and *Corynebacterium* began as primary colonizers of the NP microbiome but were in time replaced by *Dolosigranulum*, *Streptococcus*, *Moraxella* and *Haemophilus*. When evaluating the interaction between HIV exposure status and time of sampling among infants, the microbe *Staphylococcus haemolyticus* showed a distinctive high association with HIV exposure at birth. When comparing infants to their mothers with paired analyses, HIV-exposed infants’ NP microbiome composition was only slightly different from their HIV(+) mothers at birth or 14 weeks, including in their carriage of *S. pneumoniae*, *H. influenzae*, and *S. haemolyticus*.

**Conclusions:**

Our analyses indicate that the HIV-exposed infants in our study exhibit subtle differences in the NP microbial composition throughout the sampling interval. Given our results and the sampling limitations of our study, we believe that further research must be conducted in order to confidently understand the relationship between HIV exposure and infants’ NP microbiomes.

## Introduction

Currently, more than one million infants are born to women with HIV [HIV(+)] worldwide every year ^1^. Fortunately, with antiretroviral treatment for mothers and prophylaxis for their infants, the vast majority of infants with HIV exposure will not become infected with HIV ^2^. However, prevention of mother-to-child transmission (PMTCT) does not eradicate health disparities between infants who are HIV-exposed, uninfected (HIV-exposed) or HIV-unexposed, uninfected (HUU) by eliminating HIV transmission. Data suggests these children are still directly or indirectly affected by their mother’s HIV status. For example, recent meta-analyses published by members of our team reported a 60% increased risk of death ^3^ and an increased risk of pneumonia and diarrhea ^4^ among HIV-exposed compared with HUU children, thereby supporting the observed phenomenon that infants are vulnerable not only to morbidity, but also to increased mortality. These findings have also been observed in numerous other studies that have shown the linkage between poor outcomes and gastrointestinal and respiratory illnesses ^5– 9^. Hypothesized explanations include dysregulation of passive immunity via maternal antibodies, changes in the maturation of infant lymphocytes, exposure to microbes present in the mother’s birth canal at delivery, and/or social factors, which may have an impact on morbidity and mortality rates in the early stages of life.

Alternatively, it remains possible that many of these reported health differences are merely artifacts of various selection biases. Previous studies on the effects of HIV exposure were cross sectional studies based on convenience sampling and/or lacked precision due to small sample sizes ^3, 4^. Further, few such studies were longitudinal, making it difficult to observe changes over time ^3, 4^. When studying the microbiome, integral to the early development of the immune system, the lack of longitudinal structure is a crucial limitation given that it evolves dynamically over the first weeks of life, as both we and others have previously demonstrated ^10, 11^. Thus, it remains possible that much of the HIV-exposed ‘phenomenon’ of increased morbidity and/or mortality rates as currently described could be due to sociological factors, or simply selection bias. To better understand the potential biological basis for this phenomenon, unbiased, systematic data are required to build confidence in its existence.

In the gut, interactions between the microbial community and the host influence the development of the immune system and, consequently, the development of diseases ^12^. While much is known about the evolution of the intestinal microbiome, far less is known about the dynamics surrounding the microbiome of the upper respiratory tract, which plays an important role in respiratory health. If the increased rates of respiratory disease observed among HIV-exposed children have a biological explanation, we hypothesized that the respiratory microbiomes of HIV-exposed infants would differ systematically from HUU infants over the first few months of life, potentially explaining their greater susceptibility to certain respiratory diseases. Previous studies on the impact of HIV exposure have highlighted strong correlations between HIV exposure and increased risk of pneumococcal colonization and disease ^7, 13, 14^, whereas others have demonstrated no differences in pathogen carriage between children with HIV infection and control groups ^15– 17^. The current analysis seeks to address this important knowledge gap.

Given the complex dynamics of interactions between the host, microbes, and environment beginning at birth, we conducted an exploratory longitudinal comparison of the nasopharyngeal (NP) microbiomes of HIV-exposed and HUU infants and their mothers during the first 14 weeks of life. We reasoned that by characterizing quantifiable differences in NP microbiota distinguishing these two groups, this pilot study could indicate differences encouraging future immunology-focused studies.

## Methods

### Cohort characteristics

A subset of 170 NP swabs of 20 infants and their mothers were identified from a sample library collected in Lusaka, Zambia between 2015 and 2016. The sample library was part of a nested time-series case comparator study within the prospective longitudinal Southern Africa Infant-mother Pertussis study (SAMIPS) ^18^. From 1,981 total mother infant pairs, we selected a subset that had 3 or more study visits, had no siblings under the age of five years, and who enrolled in the study from April to July 2015. From this subset, we randomly selected 10 mother infant pairs with an HIV-positive [HIV(+)] mother who started antiretroviral therapy (ART) before pregnancy. These 10 mother infant pairs were randomly matched to 10 mother infant pairs with an HIV negative mother by education, month of entry into the study, and maternal age. For the cohort, infants were included if they were otherwise ‘healthy’ when screened at one week of age. Healthy infants were born at term (>37 weeks); not underweight (>2500 grams); had no acute or chronic conditions known at the time of enrollment; were not born via cesarean section; and had no known complications during pregnancy or labor and delivery. None of the mothers in the cohort experienced obstructed labor, sepsis, or hemorrhage complications during the labor and delivery period.

The institutional review boards at Boston Medical Center and Excellence in Research Ethics and Science Converge in Lusaka jointly provided ethical oversight (The ERES Converge, Lusaka. REF# 2015-Jan-002, Date: 01/02/2015; BUMC IRB, Boston. # H-33521, Date: 12/12/2014). All mothers provided written informed consent, with consent forms presented in English and the two dominant vernacular languages spoken in Lusaka: Bemba and Nyanja. The present analysis uses HIV-exposed ( *n*=10) infants and their HIV(+) mothers alongside HUU healthy control ( *n*=10) infants and their mothers who are HIV-negative [HIV(-)] collected as part of the SAMIPS study. Infant-mother pairs were recruited during their first scheduled postpartum well-child visit at approximately one week of age. Infants and their mothers were enrolled from the Chawama Primary Health Clinic (PHC) in Chawama compound, a densely populated peri-urban area near central Lusaka. Chawama PHC is the only government-supported clinic in this community and is the primary source of medical care for Chawama residents, allowing for maximal study reach. NP swabs were obtained from infants at enrollment and approximately every two to three weeks thereafter through 14 weeks of age for a total of seven scheduled time points each. Mothers’ samples were gathered at all time points at which the infants were swabbed, but only t=0 and t=6 at weeks 0–2 and 12–14 were sequenced for analysis for a total of 40 samples.

HIV(+) mothers enrolled in the SAMIPS study were required to be on ART to prevent mother-child transmission. Among the mothers in our immediate cohort, 50% (10/20) were HIV-infected, of whom 100% had initiated ART prior to conception. Given that the SAMIPS study was designed to be focused on *Pertussis* incidence, CD4 counts were not collected from study subjects, and assessment of mother’s HIV status relied upon previous testing done at the clinic. Neither viral load nor CD4 testing were routinely performed in Lusaka at this time and neither of these were deemed essential for the purposes of the original study beyond noting the mothers’ HIV status. Although data on maternal HIV status was available, final HIV status could not be ascertained on the infants themselves, which typically is not possible until the infant is four to six months of age. The study also did not collect information on breastfeeding or consumption of other foods in the study timespan, although formula is rarely used in Zambia and breastfeeding is nearly universal at such young ages ^19^. As all mothers received ART during pregnancy, pooled transmission rates of breastfeeding mothers would be about 3.54% (95% CI: 1.15–5.93%) at the six month mark ^20^. Therefore, it can be assumed that infants in this study becoming HIV(+) would be rare, and so all infants born to an HIV(+) mother were classified here as being HIV-exposed with two unknown subpopulations of HIV-exposed and HIV(+) infants. All enrolled infants received the pentavalent and pneumococcal vaccines at ages six, 10 and 14 weeks, which offer protection against 10 pneumococcal serotypes and *Haemophilus influenzae* type B. Additional information about the study structure and sampling methods can be found in Gill *et al*. (2016) ^18^.

### Sample processing and storage

NP swabs were obtained from the posterior nasopharynx using a sterile flocked tipped nylon swab (Copan Diagnostics, Merrieta, California). The swabs were then placed in universal transport media, put on ice and transferred to our onsite lab on the same campus, where they were aliquoted and stored at -80°C until DNA extraction. DNA was extracted using the NucliSENS EasyMagG System (bioMérieux, Marcy l’Etoile, France). Extracted DNA was stored at our lab located at the University Teaching Hospital in Lusaka at -80°C. Sample collection, processing and storage were previously described (Gill *et al*., 2016) ^18^.

### 16S ribosomal DNA amplification and MiSeq sequencing

For 16S library preparations, two PCR reactions were completed on the template DNA. Initially the DNA was amplified using universal bacterial primers ^21^ specific to the V3–V4 region of the 16S rRNA gene ^21^. Library preparation was performed according to the standard instructions of the 16S Metagenomic Sequencing Library Preparation protocol (Illumina, USA). The 16S primer pairs incorporated the Illumina overhang adaptor (16S forward primer 5’-TCGTCGGCAGCGTCAGATGTGTATAAGAGACAGCCTACGGGNGGCWGCAG-3’; 16S reverse primer 5’-GTCTCGTGGGCTCGGAGATGTGTATAAGAGACAGGACTACHVGGGTATCTAATCC-3’)

Each PCR reaction contained DNA template (~12 ng), 5µℓ forward primer (1μM), 5 µℓ reverse primer (1μM), 12.5 µℓ 2 X Kapa HiFi Hotstart ready mix (KAPA Biosystems Woburn, MA), and PCR grade water to a final volume of 25µℓ. PCR amplification was carried out as follows: heated lid 110°C, 95°C for 3 min, 25 cycles of 95°C for 30s, 55°C for 30s, 72°C for 30s, then 72°C for 5 min and held at 4°C. Negative control reactions without any template DNA were carried out simultaneously.

The size of the amplicons was then visualized using the 4200 TapeStation (Agilent Technologies, Germany). Successful PCR products were cleaned using AMPure XP magnetic bead-based purification (Beckman Coulter, IN). The IDT for Illumina Nextera DNA UD Indexes kit (Illumina, San Diego, CA) with unique dual index adapters were used to allow for multiplexing. Each PCR reaction contained purified DNA (5 μℓ), 10 μℓ index primer mix, 25 μℓ 2X Kapa HiFi Hot Start Ready mix and 10 μℓ PCR grade water. PCR reactions were performed on a Bio-Rad C1000 Thermal Cycler (Bio-Rad, Hercules, CA) Cycling conditions consisted of one cycle of 95°C for 3 min, followed by eight cycles of 95°C for 30 s, 55°C for 30 s and 72°C for 30 s, followed by a final extension cycle of 72°C for 5 min. PCR products of negative controls were confirmed negative on Agilent TapeStation (no band observed).

Prior to library pooling, the indexed libraries were purified with Ampure XP beads and quantified using the Qubit dsDNA HS Assay Kit (Thermo Fisher Scientific, Waltham, MA). Purified amplicons were run on the Agilent TapeStation (Agilent Technologies, Germany) for quality analysis before sequencing. The sample pool (2 nM) was denatured with 0.2N NaOH, then diluted to 4 pM and combined with 10% (v/v) denatured 20 pM PhiX, prepared following Illumina guidelines. Libraries were then sequenced on the Illumina MiSeq sequencing platform (Illumina, USA) at the Sequencing Core Facility, National Institute for Communicable Diseases (NICD) of the National Health Laboratory Service, South Africa, using a 2 x 300 cycle V3 kit, following standard Illumina sequencing protocols. Negative controls were sequenced as well, resulting in extremely low reads that were not further analyzed.

In addition to using negative controls, all samples were processed at random to account for reagent contamination. Lab technicians were blinded to the timing of sample collection and clinical data.

We assessed the quality of the sequencing data using FastQC v0.11.9 ^22^. Trimmomatic ^23^ v0.39 was used to trim Illumina adapters and remove low-quality sequences. We performed a sliding window trim, cutting once when the average quality score within a window of six bases falls below 15. We removed both leading and trailing low quality or N bases below quality six. All other parameters used the default settings.

Sequencing data were aligned to bacterial genomes and profiled using the PathoScope 2.0 pipeline ^24^. All RefSeq representative bacterial genomes available as of November 2, 2018 were used as a PathoScope reference library. We obtained target read counts delineated by National Center for Biotechnology Information (NCBI) unique identifiers (UIDs) and matched them to the NCBI Taxonomy database to retrieve accurate taxonomic hierarchy information. We then aggregated reads by genera. Data were transformed to relative abundances using counts per million (CPM) and normalized using log CPM for subsequent analyses. In most cases, taxa belonging to genera with average relative abundances of less than 1% were grouped as “Other” in analyses. All code has been made available via Zenodo ^25^.

### Visualizing longitudinal trends in NP microbiome composition

Microbial abundances across sample groups were visualized using alluvial diagrams and stacked bar plots using the R packages ggplot2 v3.3.5 and alluvial v0.2-0. The alluvial diagrams illustrate individual genera as stream fields that change position at different time points. The height of a stream field represents the relative abundance of that taxon. At a given time point, stream fields are ranked from the highest to lowest abundance (top to bottom). These were plotted for infants according to HIV exposure status over several time points. Stacked bar plots were used to visualize the relative abundance of microbes at a given taxonomic level in each sample, represented as a single bar, labeled by time point, and plotted within each HIV exposure status group for separate mothers and infant comparisons. These plotting and diagramming techniques allow for an efficient overview of the types of differences inherently present in the data at the group level.

### Modeling effects of HIV status on infant NP microbiome composition across time

Generalized estimating equations (GEEs) as described in Liang and Zeger (1986) ^26^ and extended by Agresti (2002) ^27^ have been widely used for modeling longitudinal data ^28^, and more recently for longitudinal microbiome data ^29, 30^. For each genus present in the microbial aggregate of samples, we modeled normalized log CPM relative taxon counts, estimating the effects of time point and HIV exposure status and their interaction, while accounting for the underlying structure of clusters formed by individual subjects. We defined the responses *Y*_1_, *Y*_2_,..., *Y _n_* as the collection of infant relative abundances for a given taxon in log CPM, with *n* = 129. We identified the mean model *µ _ij_* for the *i ^th^* infant and *j ^th^* timepoint. With regression parameters *β _k_* representing HIV exposure status and time point, and the AR(1) variance structure *V _i_*, we formed the estimating equation:

$$U(\beta )=\sum_{i=1}^{n}\frac{\partial \mu_{i}}{\partial \beta }\;V_{i}^{-1}\{Y_{i}-\mu_{i}(\beta )\}.$$

This then becomes an optimization problem, such that solving *U*( *β*) = 0 estimates the parameters *β _k_*. We modeled abundances for all 12 genera and nine of the top species. The link function *g* was chosen to be a Gaussian link. It was assumed that these abundances are correlated within infants for the various sampling time points. As such, we accounted for this with a first-order autoregressive AR(1) working correlation structure with homogenous variances such that the correlation between adjacent time points was assumed to be more similar. Parameter estimates were collected from each model, along with Wald test p-values. A Bonferroni correction was applied to account for multiple hypothesis testing, with an initial α=0.05. Due to the conservative nature of the Bonferroni correction, adjusted p-values between 0.05 and 0.10 were noted as marginally significant as effects that could retain some level of practical significance. Models were created in R using geepack v1.3.10 ^31^.

### Testing differences in NP microbiome composition across time and cohort groups

Hotelling’s *T* ^2^ tests ^32^ were used to determine whether the microbiome profiles exhibited notable differences or trends across time and groups. Student’s t-tests were used to identify which genera contributed most to these differences. Groups of mothers and infants or HIV-exposed and HUU infants are designated as the two sampling units on which the relative abundances of the *p* most abundant genera were measured. For paired tests, we chose *p* = 6 variables to ensure that *n* < *p* so that singularity could be avoided and *T* ^2^ could be properly computed, where *n* is the number of measurements in a sampling unit. We tested the hypotheses

$$H_{0}:\mu_{y}=\mu_{x}\;\text{vs.}\;H_{A}:\mu_{y}\neq \mu_{x}$$

which, in the paired case given *μ _diff_* = *μ _y_* – *μ _x_*, is equivalent to

$$H_{0}:\mu_{diff}=0\;\text{vs.}\;H_{A}:\mu_{diff}\neq 0$$

to conduct a comparison between groups for the *p* most abundant genera for testing groups. The population means *μ _x_* and *μ _y_* represent the two sampling units of a given test, which were generally either *μ _mothers_* and *μ _infants_* or *μ _HUU_* and *μ _HEU_*. We treated samples as paired in both cases such that the mothers are paired with their own infants or HIV-exposed and HUU infants were paired according to the pre-analysis matching schema. When testing HIV positive mothers versus HIV negative mothers, we did not pair mothers and instead relied on a standard two-sample test. We assumed that the relevant conditions for testing are met, meaning that the groups are correlated and have a multivariate normal distribution. Normality assumptions are met by using microbe abundances in log CPM units.

We conducted three main groups of testing with the *T* ^2^ statistic. The first paired test was between infant-mother pairings at the first or last time point ( *t* ∈ [0, 6]) and for either the infant/mother HIV-exposed/HIV(+) or HUU/HIV(-) groups for a total of four tests. These tests offer a clear between-group comparison while accounting for similarity in the infant-mother dyads and calculating genera-specific differences in ruling whether the hypotheses are met. The second testing approach consisted of seven tests. We compared relative abundances of the six most highly abundant genera at each of the seven time points ( *t* ∈ [0, 1,..., 6]) from paired HIV-exposed and HUU infants. The final testing approach compared all HIV(+) and HIV(-) mothers’ samples for all 12 genera across mothers, including the “Other” designation as described previously. All samples were unpaired and therefore utilized the unpaired multivariate *T* ^2^ generalization.

Test statistics were calculated to test whether microbiome profiles in paired infants were notably different among the seven time points, and whether HIV-exposed mothers and infants had similar profiles at t=0 and t=6. After calculating the *T* ^2^ test statistic, we calculate an equivalent *F _p,n–p_* test statistic distributed with *p* and *n* – *p* degrees of freedom and use this distribution to calculate the test’s p-value:

$$\text{p-value}=\text{Pr}\left\{F_{p,n-p+1}>\frac{n-p+1}{np}T^{2}\right\}$$

Paired or two-sample t-tests were used to distinguish which genera are most important to the differences identified with Hotelling’s *T* ^2^ tests, and *α*-levels were adjusted after performing the *p* tests by using a Bonferroni critical value to reduce Type I error rate inflation.

Beta diversity using the Bray-Curtis dissimilarity metric was compared using a non-parametric Wilcoxon rank-sum test between (1) HIV-exposed and HUU infants, and (2) between HIV(+) and HIV(-) mothers. Tests were then conducted separately for each subset of samples for (3) t=0 and (4) t=6 for a total of four tests. The null hypothesis conjectures that the distributions of both populations are equal under the general assumption that all observations from both groups are independent of each other.

All analyses were performed using R Statistical Software (v4.2.1; R Core Team 2022).

## Results

### Sample characteristics

Characteristics of the study cohort are delineated in Table 1.

**Table 1.** Baseline demographic characteristics of the full infant cohort, stratified by HIV exposure status. IQR refers to the interquartile range, BCG refers to the Bacille Calmette-Guérin vaccine for tuberculosis disease, and OPV refers to oral polio vaccine.

Parameter	HIV-Exposed	HIV-Unexposed	All Subjects
**Infant Demographics**			
Number of Infants	10	10	20
Male Sex % (n)	50.0% (5/10)	40.0% (4/10)	45.0% (9/20)
Median Age in Weeks at Enrollment (IQR)	0.86 (0.86–1)	0.93 (0.86–1)	0.86 (0.86–1)
Median Estimated Gestational Age at Delivery (weeks) (IQR)	40.0 (38–40)	40.0 (39–40)	40.0 (39–40)
Median Birth Weight (IQR)	3050.0 (2800–3200)	3100.0 (3000–3100)	3100.0 (2900–3200)
**Maternal Demographics**			
Number of Mothers	10	10	20
Median Age in Years at Enrollment (IQR)	34.0 (29–36)	34.0 (30–37)	34.0 (30–37)
Married % (n)	100.0% (10/10)	100.0% (10/10)	100.0% (20/20)
**Immunizations at birth**			
BCG % (n)	30.0% (3/10)	50.0% (5/10)	40.0% (8/20)
OPV % (n)	30.0% (3/10)	20.0% (2/10)	25.0% (5/20)
**Labor and Delivery Complications**			
Birth Asphyxia % (n)	10.0% (1/10)	0.0% (0/10)	5.0% (1/20)
**Maternal Immunization**			
Median Number of Tetanus Toxoid Doses (IQR)	5.0 (3–5)	5.0 (3–5)	5.0 (3–5)
**Maternal HIV Status**			
Mother HIV(+) % (n)	100.0% (10/10)	0.0% (0/10)	50.0% (10/20)
Mother on ART % (n)	100.0% (10/10)	. % (./0)	100.0% (10/10)
Mother on ART Prior to Pregnancy % (n)	100.0% (10/10)	. % (./0)	100.0% (10/10)
**Place of Birth**			
UTH % (n)	40.0% (4/10)	30.0% (3/10)	35.0% (7/20)
Chawama Clinic % (n)	60.0% (6/10)	60.0% (6/10)	60.0% (12/20)
Home Delivery % (n)	0.0% (0/10)	10.0% (1/10)	5.0% (1/20)
**Household composition**			
Median Household Size (IQR)	5.5 (4–7)	6.0 (5–7)	6.0 (5–7)

Some missingness in the data is present; for ten samples, these visits either never occurred or swabs were not collected. An additional three samples were excluded from analysis because fewer than 10,000 reads aligned to RefSeq reference genomes. This left us with 129 infant samples and 38 mother samples for analysis. In total, 16/20 infants had data for all seven time points, 2/20 infants had only six time points, 1/20 infants had three time points, and 1/20 infants had only two time points. All infants had swabs for t=0 and t=1. Two of 20 mothers lacked swabs at t=6 ( Table 2).

**Table 2.** Counts and percentages of samples available for analysis from both infants and mothers. These are stratified by time point, infant age in weeks, and HIV status.

Time point	HUU Infant	HIV(-) Mother	HIV-Exposed Infant	HIV(+) Mother	Overall
	(N=68)	(N=19)	(N=61)	(N=19)	(N=167)
0	10 (14.7%)	10 (52.6%)	10 (16.4%)	10 (52.6%)	40 (24.0%)
1	10 (14.7%)	0 (0%)	10 (16.4%)	0 (0%)	20 (12.0%)
2	10 (14.7%)	0 (0%)	9 (14.8%)	0 (0%)	19 (11.4%)
3	9 (13.2%)	0 (0%)	8 (13.1%)	0 (0%)	17 (10.2%)
4	9 (13.2%)	0 (0%)	8 (13.1%)	0 (0%)	17 (10.2%)
5	10 (14.7%)	0 (0%)	8 (13.1%)	0 (0%)	18 (10.8%)
6	10 (14.7%)	9 (47.4%)	8 (13.1%)	9 (47.4%)	36 (21.6%)

Although we included 10 HUU and 10 HIV-exposed infants and their mothers in the study, there was some small variation in numbers of samples at the different time points, but they were overall close in evenness. Infants were approximately 1 ± 0.14 (mean ± SD) weeks old at time of first swabbing and were on average 3.14 ± 0.28 weeks, 6 ± 0.42 weeks, 8.29 ± 0.29 weeks, 10.57 ± 0.29 weeks, 12.57 ± 0.29 weeks, and 15 ± 0.29 weeks respectively at subsequent time points t=1 through t=6.

Our FastQC analysis indicated that the overall sequencing quality was excellent, with mean Phred quality scores remaining greater than 25 (99.5% accuracy) for at least 175 bp for both forward and reverse reads. Trimmomatic removed less than 3% of reads in any given sample. The analysis covered 129 infant and 38 mother swab samples, with an average of 124,905 ± 299,120 (mean ± SE) reads per infant sample (max = 3,016,276; min = 13,218) and 70,463 ± 48,730 reads per mother sample (max = 208,029; min = 14,339). The read count for infant samples was significantly higher than that of mothers’ swabs (p = 0.02), which may in part be the result of mothers’ acquired immunity over time and therefore lower overall NP carriage. In our raw data, we uniquely identified 17 phyla, 647 genera, and 758 total species across all samples. Due to the high diversity of taxa with relatively low abundance among individual microbes, we labeled taxa with average relative abundances of less than 1% as “Other” at the genera and species level. Post-grouping, the present taxa were limited to three distinctly identifiable phyla, encompassing 12 genera and 87 species. The most abundant phyla were the Firmicutes (~56.5%), Proteobacteria (~22.4%), and Actinobacteria (~16.1%). The remaining 5% of reads were characterized as “Other.” At the genus level, the most abundant groups were the *Dolosigranulum* (~23.5%), *Staphylococcus* (17.2%), *Corynebacterium* (~16.1%), *Streptococcus* (~13.5%) and *Moraxella* (~12.4%) genera.

### Visualizing longitudinal trends in NP microbiome composition

**Figure 1.**
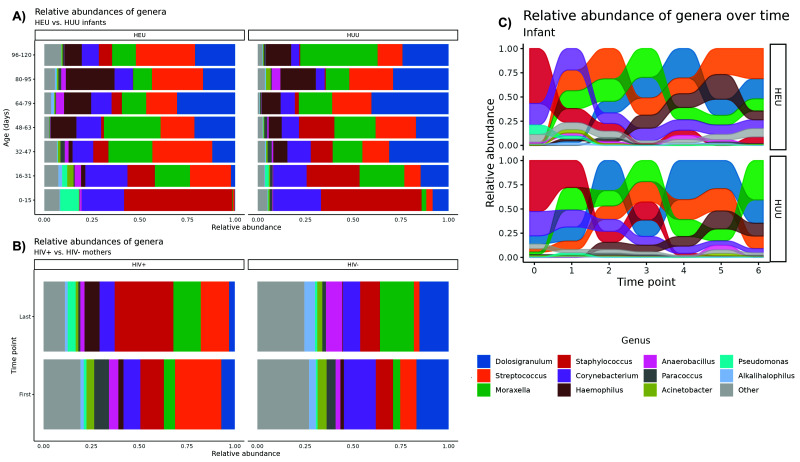
The maturation over 17 weeks of the NP microbiomes of **A**) healthy HIV-exposed (n=10) and HUU infants (n=10) and **B**) HIV(+) (n=10) and HIV(-) mothers (n=10). These stacked bar plots reveal variation in the relative abundance of microbes between groups of either infants or mothers clustered at the genus level. Each bar represents a single time point binned by age and is the average of ~10 samples. Genera with an average relative abundance of <1% across all samples are labeled as “Other.” The alluvial plot in **C**) depicts the changing presence of genera across all infant samples by HIV exposure status. As relative abundances change over time, the position of a flow stream representing a single genus may change position relative to the other genera.

Longitudinal trends in NP microbiome composition by HIV exposure status are depicted in Figures 1A and 1B for infants and mothers, respectively. The alluvial plot in Figure 1C graphically depicts the most abundant genera in log CPM present in HIV-exposed and HUU infants. As relative abundances (in log CPM) change over time, the position of a flow stream representing a single genus may change position relative to the other genera. At a given time point, the flow streams are stacked according to the abundance relative to other streams.For both the HEU and HUU infant groups Across all infant samples, the respiratory microbiome during the first months of life is dominated by *Staphylococci* and *Corynebacteria.* Early on, we observed the emergence of more typical respiratory bacteria such as *Moraxella* and *Streptococcus* sp. Additionally, the commensal *Dolosigranulum* emerges as a dominant member of the microbiome within the first weeks of life. *Haemophilus* appeared later at around four to six weeks.

### Effect of HIV status on infant NP microbiome composition across time

While longitudinal trends were strongly apparent across infant groups, the observed differences between the HIV-exposed and HUU infants were subtle. Figure 1A illustrates higher amounts of *Streptococcus* and suppression of *Dolosigranulum* among HIV-exposed vs. HUU infants. Furthermore, Figure 1C suggests increasing amounts of *Streptococcus* and *Haemophilus* over time for HIV-exposed infants, whereas *Staphylococcus* largely declines after t=3 in both groups.

**Figure 2.**
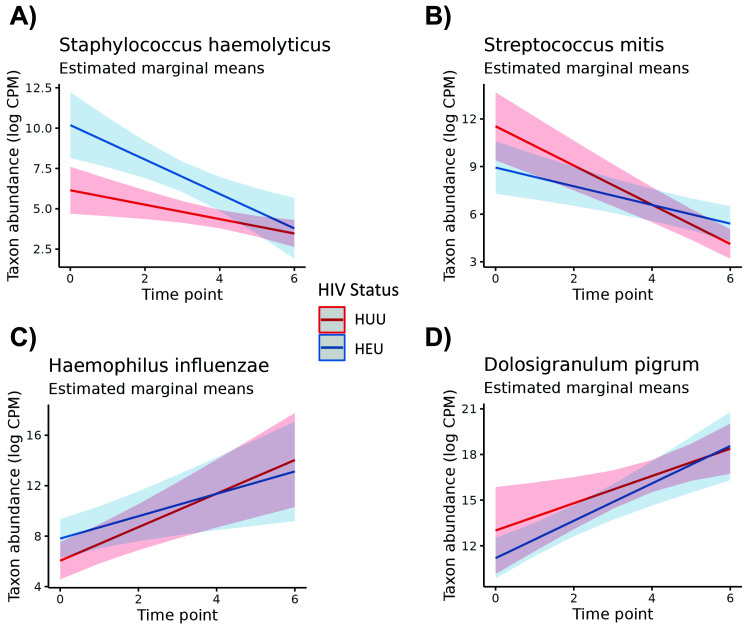
Plots of estimated marginal means of the abundance of a given microbe, given the estimated GEE effects of time point of sampling (x axis) and HIV status (line color). A line illustrates the estimated change in microbe abundance over time, with positive or negative slopes illustrating increased or decreased estimated abundance in log CPM (respectively). HIV-exposed infants (denoted as HEU) are depicted in blue, and HIV-unexposed, uninfected infants (HUU) are depicted in red. Given the separate lines for each status and changing slope across time, these plots depict the interaction effect between time point and HIV status. All microbes had at least marginally significant time effects, but only *S. haemolyticus* had a very strong HIV exposure status effect.

GEEs revealed some differences among genera and species for time point and HIV exposure status when adjusting for subject variation and the interaction effect. Models were created for all 12 genera, including “Other” genera, and for the top nine species that averaged greater than 1% abundance across all infant samples. Of all taxa tested, four of nine species and six of 12 genera exhibited substantial instability across time points ( Table 3). One species, *Staphylococcus haemolyticus*, was highly associated with HIV exposure in infants (p <0.01, adj. p = 0.01). Additionally, *Streptococcus mitis* indicated a strong interaction effect between time and HIV exposure status (p <0.01, adj. p = 0.04). Figure 2 shows the estimated marginal means of the time and HIV-exposure status effects for *S. haemolyticus*, *S. mitis*, *Haemophilus influenzae*, and the *Dolosigranulum* genus. We chose to include these microbes in the figure as a representative selection of microbes with significant and non-significant effects. In Figure 2a, differences in the abundance of *S. haemolyticus* for HIV-exposed and HUU infants is cleanly pronounced for several time points, reflecting the Wald test result, but this is not the case for the other microbes in Figures 2b, 2c, or 2d. All of these had at least marginally significant p-values when testing the time point effect but lacked strong HIV exposure status effects.

**Table 3.** Table of P-values from Wald tests on GEE models. The effects modeled were HIV status, and time point effects, with their interaction. Results are stratified by species and genus. All p-values less than alpha=0.05 are bolded.

	Time point Coefficient Estimate	Time point Unadjusted p-values	Time point Adjusted p-values	HIV Status Coefficient Estimate	HIV Status Unadjusted p-values	HIV Status Adjusted p-values	Interaction Coefficient Estimate	Interaction Unadjusted p-values	Interaction Adjusted p-values
**Genus**									
*Acinetobacter*	-0.09	**0.00**	**0.02**	-0.06	0.78	1.00	0.04	0.29	1.00
*Alkalihalophilus*	0.18	**0.01**	0.07	0.67	0.15	1.00	-0.26	**0.03**	0.30
*Anaerobacillus*	-0.11	0.19	1.00	0.77	0.12	1.00	-0.06	0.56	1.00
*Corynebacterium*	-0.18	**0.01**	0.09	-0.39	0.11	1.00	0.12	0.18	1.00
*Dolosigranulum*	0.25	**0.01**	0.13	-0.46	0.35	1.00	0.09	0.46	1.00
*Haemophilus*	0.43	**0.00**	**0.00**	0.69	0.14	1.00	-0.18	0.23	1.00
*Moraxella*	0.35	**0.00**	**0.00**	0.54	0.21	1.00	-0.14	0.07	0.89
*Other*	-0.09	**0.03**	0.30	-0.04	0.81	1.00	0.06	0.25	1.00
*Paracoccus*	-0.04	0.44	1.00	-0.50	0.08	0.99	0.12	0.09	1.00
*Pseudomonas*	-0.15	**0.00**	**0.00**	-0.52	0.19	1.00	0.17	**0.03**	0.40
*Staphylococcus*	-0.38	**0.00**	**0.00**	-0.13	0.53	1.00	0.04	0.35	1.00
*Streptococcus*	0.13	**0.00**	**0.01**	-0.18	0.43	1.00	0.09	0.12	1.00
**Species**									
*Corynebacterium accolens*	-0.27	0.52	1.00	-2.34	0.26	1.00	0.70	0.18	1.00
*Corynebacterium pseudodiphtheriticum*	0.25	0.58	1.00	-1.91	0.30	1.00	0.51	0.34	1.00
*Dolosigranulum pigrum*	0.90	**0.01**	0.06	-1.82	0.26	1.00	0.33	0.42	1.00
*Haemophilus influenzae*	1.33	**0.00**	**0.00**	1.76	0.11	0.96	-0.44	0.30	1.00
*Staphylococcus epidermidis*	-0.57	**0.03**	0.24	-0.07	0.97	1.00	-0.21	0.56	1.00
*Staphylococcus haemolyticus*	-0.45	**0.01**	0.07	4.04	**0.00**	**0.01**	-0.62	0.07	0.59
*Staphylococcus simiae*	-1.27	**0.00**	**0.01**	-4.49	0.11	1.00	0.88	0.08	0.69
*Streptococcus mitis*	-1.24	**0.00**	**0.00**	-2.60	0.06	0.53	0.65	**0.00**	**0.04**
*Streptococcus pneumoniae*	1.81	**0.00**	**0.00**	1.85	0.16	1.00	-0.20	0.52	1.00

Hotelling’s *T* ^2^ test statistics were used to compare relative abundances of the top six most abundant genera between paired HIV-exposed and HUU infants at each time point, namely *Dolosigranulum*, *Streptococcus*, *Moraxella*, *Staphylococcus*, *Corynebacterium*, and *Haemophilus*. Paired infants did not present significantly different microbiome profiles at any of the time points at *α* = 0.05 ( Table 4). The largest differences seemed to occur at t=5 (p = 0.07).

**Table 4.** Table of Hotelling’s T ^2^ test results. Tests compared log CPM relative genera abundances of paired HIV-exposed and HUU infants at all seven time points. The first and second degrees of freedom for the test are denoted by *df*_1_ and *df*_2_, respectively.

	t=0	t=1	t=2	t=3	t=4	t=5	t=6
*df*_1_	6	6	6	6	6	6	6
*df*_2_	4	4	3	1	1	2	2
**Critical *F* value**	6.16	6.16	8.94	233.99	233.99	19.33	19.33
***F* statistic**	0.90	1.99	0.47	3.41	22.66	14.56	1.26
**p-value**	0.57	0.26	0.80	0.39	0.16	0.07	0.50

We examined variations in overall microbiome composition by observing differences in beta diversity between HUU and HIV-exposed infants. We conducted two Wilcoxon rank-sum tests of the Bray-Curtis dissimilarity at t=0 and t=6. We found a non-significant difference in microbiome composition between HUU and HIV-exposed infants at t=0 (p = 0.68), but large differences at t=6 (p <0.01). Performing additional tests within the HIV-exposed and HUU groups themselves, we also identified heavy inter-subject variability at both t=0 and t=6 (all p-values < 0.01). As a result of this inter-subject variability, alpha diversity metrics were not performed for this analysis.

### Associations within mother and infant duad NP microbiome composition over time

We also investigated the NP microbiome relationship within infant-mother duads. Paired Hotelling’s *T* ^2^ tests were used to test the log CPM of genera as a measure of relative abundance at t=0 and t=6 for the HIV-exposed/HIV(+) and HUU/HIV(-) groups ( Table 5). Genera tested were the same as listed for the infant-specific Hotelling’s tests. HIV-exposed/ HIV(+) infant-mother pairs had marginally significant profile differences at t=0 ( $T_{6,4}^{2};$ p = 0.10) but were similar enough to not present as having significant profiles at t=6 ( $T_{6,1}^{2};$ p = 0.60). HUU/HIV(-) infant-mother pairs also had marginally significant differences at the t=0 ( $T_{6,4}^{2};$ p = 0.10) but varied notably at t=6 ( $T_{6,3}^{2};$ p = 0.02). Paired t-tests for the six genera were separately conducted for HUU/HIV(-) infant-mother pairs to distinguish which genera are most important to the identified difference at that time point. The largest difference in abundance occurred for the *Haemophilus* ( *t*_8_, p = 0.02; adj. p-value = 0.10) and *Staphylococcus* genera ( *t*_9_; p-value <0.01; adj. p-value <0.01). HUU Infants were noted as having greater *Haemophilus* carriage than their HIV(-) mothers, whereas mothers had greater *Staphylococcus* carriage than their infants.

**Table 5.** Table of Hotelling’s T ^2^ test results. Tests compared log CPM relative genera abundances at t=0 and t=6 for the HIV-exposed/HIV(+) and HUU/HIV(-) groups using infant-mother pairs.

	Time point t=0		Time point t=6	
	HIV-exposed/HIV(+)	HUU/HIV(-)	HIV-exposed/HIV(+)	HUU/HIV(-)
*df*_1_	6	6	6	6
*df*_2_	4	4	1	3
**Critical *F* value**	6.16	6.16	233.99	8.94
***F* Statistic**	4.13	4.14	1.20	19.52
**p-value**	0.10	0.10	0.60	**0.02**

Although all infants in our cohort were free from illness, we found that certain pathogenic species were present in modest abundance across samples. To ascertain the possibility of HIV-exposed infants acquiring pathogens from their HIV(+) mothers, we conducted paired t-tests for three well-known pathogenic species: *Streptococcus pneumoniae*; *Haemophilus influenzae*; and *Staphylococcus haemolyticus*. Tests compared pairs at the t=0 and t=6 time points and within either HIV(+) or HIV(-) subgroups for a total of four different tests per species ( Table 6). Inequality in pathogen carriage was ascertained by the mean of differences in log CPM; positive values indicate higher abundance in mothers. *H. influenzae* was more abundant in HUU infants than in their HIV(-) mothers at t=6 (adj. p = 0.1), and *S. haemolyticus* was likely to be found in the HUU infants at t=0 in a higher concentration than in their HIV(-) mothers (adj. p = 0.1). *S. pneumoniae* was more likely to be found in HUU infants than their HIV(-) mothers at t=6 (adj. p = 0.1). Each of these findings were marginally significant.

**Table 6.** Results table from paired t-tests of log CPM relative abundances of well-known pathogenic species. Tests compared infant-mother pairs at the first and last time points, and within either HIV or control subgroups, for a total of four different tests per species. Lower and upper 95% confidence interval bounds are denoted by the Lower CI and Upper CI columns. A positive mean of differences value indicates higher abundance of a pathogen in mothers. A Bonferroni correction was applied to account for multiple hypothesis testing (see adjusted p-values).

Species	HIV Status	Time point	Mean of differences (log CPM)	Lower CI	Upper CI	p-value	Adjusted p-value
*Streptococcus pneumoniae*	HIV-exposed/HIV(+)	0	2.03	0.26	3.80	**0.03**	0.4
		6	-1.37	-3.47	0.73	0.16	1.0
	HUU/HIV(-)	0	1.42	-0.43	3.28	0.12	1.0
		6	-2.83	-4.86	-0.79	**0.01**	0.1
*Haemophilus influenzae*	HIV-exposed/HIV(+)	0	0.16	-1.55	1.87	0.84	1.0
		6	-1.08	-3.87	1.70	0.38	1.0
	HUU/HIV(-)	0	0.09	-1.18	1.36	0.88	1.0
		6	-1.57	-2.69	-0.45	**0.01**	0.1
*Staphylococcus haemolyticus*	HIV-exposed/HIV(+)	0	-2.31	-4.36	-0.27	0.03	0.4
		6	0.56	-0.94	2.06	0.40	1.0
	HUU/HIV(-)	0	-1.81	-3.02	-0.61	**0.01**	0.1
		6	0.54	-0.52	1.60	0.27	1.0

### Effect of HIV status on mother NP microbial community composition

We compared HIV(+) and HIV(-) mothers at the t=0 and t=6 time points. We used the Bray-Curtis dissimilarity metric as a measure of beta diversity to compare compositional dissimilarity between NP microbiomes. We identified strong dissimilarity between HIV(+) and HIV(-) mothers at t=6 (p <0.01), but higher similarity at t=0 (p = 0.70). Additionally, the summed abundances of all twelve genera, including “Other” genera, were used to conduct a two-sample Hotelling’s *T* ^2^ test on unpaired mothers. The differences we observed were more noticeable at the latter ( $T_{12,5}^{2};$ p = 0.04). Unpaired *t*-tests indicated that the “Other” taxa (p = 0.03, adj. p = 0.37), *Alkalihalophilus* (p = 0.08, adj. p = 1), and *Paracoccus* (p = 0.10, adj. p=1) were the largest contributors to this difference. These taxa were all more highly abundant in the HIV(-) mothers.

## Discussion

Our study explored differences in the NP microbiome among Zambian HIV-exposed and HUU infants and their mothers over a three-month period. Our starting point for this analysis was to better understand the observed excess mortality and increased rates of respiratory disease among HIV-exposed infants. Assuming that such differences are not merely due to sampling biases, the chief hypotheses explaining them are that 1) HIV-exposed infants have subtle immunological deficits, or 2) these are the consequence of confounding due to environmental and sociological factors. These are not mutually exclusive, and we acknowledge that all drivers are likely inter-related and difficult to differentiate. We reasoned that they may be in turn associated with differences in the microbiota which could then serve as a quantifiable indicator of differences in respiratory health between HIV-exposed and HUU infants. Our analyses indicate that the HIV-exposed infants in our study exhibited subtle differences in the NP microbial composition throughout the sampling interval. Given our limited number of samples, it is within reason that these differences are a result of sample variation. Although we cannot exclude true differences between the populations, we are left with uncertain evidence that there is an HIV exposure effect on the NP microbiome in the first 14 weeks after birth.

To date, many studies have examined the effect of the microbiome of HIV(+) mothers on their infants and found noticeable differences. Bender *et al.* (2016) reported that although very few differences were apparent in the microbiomes of mothers with and without HIV infection, maternal HIV infection was associated with changes in the mouth, skin, and gut microbiome of HIV-exposed infants from Haiti ^33^. Higher abundance of *Pseudomonadaceae* and *Thermaceae*, along with decreased bacterial diversity in stools of HIV-exposed infants was suggested as one mechanism that accounts for the immunologic derangements and poor growth observed in these children. It has been noted that the microbiome of mother and infant dyads reveals some associations with HIV infection ^34^, particularly that infants’ microbiomes reflect the dysbiosis of their mothers, but how this dysbiosis is established in the HIV-exposed infant is poorly understood. Significantly higher bacterial diversities have been found in the fecal matter of HIV-exposed infants, compared to HIV-unexposed infants in an African cohort ^35^. The relatively small number of studies looking specifically at the microbiome of the nasopharynx in HIV-exposed infants have found few changes associated with HIV infection ^36^, even though increased risk of pneumococcal colonization and disease remains apparent for these infants ^7, 13, 14^. Although dysbiosis seems to be a reasonable factor in this risk, the few available data contradict this hypothesis, demonstrating no differences in pathogen carriage between children with HIV infection and control groups ^15– 17^.

### Longitudinal trends in infant NP microbiome composition

Initial plots of the microbial relative abundances appeared to show dynamic changes in abundances of certain genera; it is apparent from Figure 1A and 1C that *Staphylococcus* and *Corynebacterium* began as primary colonizers of the NP microbiome but were replaced by *Dolosigranulum*, *Streptococcus*, *Moraxella* and *Haemophilus* over time. Overall, these transitions occurred in an orderly and stepwise pattern over time. These transitional patterns align with those found in a longitudinal East Asian infant cohort ^37^.

Given the few studies conducted on the NP microbiome in Zambia, there are no conclusive baseline expectations for a typical microbiome composition. However, we have compiled the results of a published longitudinal study that analyzed NP swabs from a cohort of 234 healthy infants from Washington, D.C. ^38^ at ~two, ~six, and ~12 months ( Table 7). Slight differences appear to be present, which may be in part due to differences in living conditions, sample size, and choice of analysis database used. The Teo *et al.* study utilized the Greengenes database, which produces far less sensitive results on 16S amplicon sequencing data when compared to other databases such as RefSeq and Silva ^39^. Overall, *Corynebacterium*, *Moraxella*, *Streptococcus* averages seem to be quite similar, with stark differences in *Staphylococcus* and *Dolosigranulum abundances*. While this study is not directly comparable to the Teo *et al.* study, it appears that the NP microbiome profiles observed here are similar to those seen in healthy infants of a similar age.

**Table 7.** Comparison of average genera relative abundance with an NP longitudinal study of a healthy infant cohort from Washington, D.C. The number of samples, age group by month (m), sequenced 16S region, patient condition and genus mean relative proportions are enumerated. While the studies are not a one-to-one comparison, the relative abundances between studies appear to be similar. NR denotes statistic not reported in original paper.

Study	Teo *et al*. (2015) ^32^	Teo *et al*. (2015) ^32^	This study	This study
Number of samples	1,021	~177	68	61
Age group	2–12 m	2 m only	0–3.5 m	0–3.5 m
16S Region	V4	V4	V3–V4	V3–V4
Patient condition	Healthy	Healthy	Healthy	HIV-exposed
**Microbe**				
*Dolosigranulum*	8.8%	14.0%	32.0%	12.4%
*Streptococcus*	14.0%	14.0%	8.9%	14.5%
*Moraxella*	31.2%	9.0%	10.7%	15.7%
*Staphylococcus*	10.3%	41.0%	19.7%	14.5%
*Corynebacterium*	13.5%	22.0%	17.9%	14.9%
*Haemophilus*	9.7%	NR	4.9%	9.2%
*Anaerobacillus*	NR	NR	0.9%	2.0%
*Paracoccus*	NR	NR	1.2%	0.6%
*Acinetobacter*	13.0%	NR	1.1%	0.7%
*Pseudomonas*	NR	NR	0.3%	3.6%
*Alkahilophilus*	NR	NR	0.2%	0.7%
Other	NR	NR	2.2%	5.6%

### Effect of HIV status on infant NP microbiome composition across time

Our plots appear to show *Dolosigranulum* may have higher carriage with simultaneous lower carriage of *Streptoccocus* in HUU when compared to HIV-exposed infants. Some of these observations are supported by the GEE findings ( Table 3). For instance, some taxa appeared to be present in larger proportions at certain time points for HIV-exposed or HUU infants, but in general, taxa seemed to follow the same general trends in both groups. Regardless of HIV exposure status, it was apparent that as the child grows, an increasing amount of respiratory flora emerges. As time went on, we found that HIV-exposed infants diverged in their microflora profiles and diversity from the HUU infants, as was shown by the Wilcoxon test result at t=6. Interestingly, this was not verifiable in the multivariate Hotelling’s T-squared test that collectively tested the top six of the most prevalent genera over time. This indicates that differences may have occurred for individual genera at a given time point yet did not result in holistic trends involving several of the top genera. The largest difference seemed to occur at t=5. It would seem reasonable to suggest that at birth, infants’ microbiome profiles are more similar than different, and that the diversification of these profiles occurs over time; however, the lack of any considerable difference at t=6 disputes this argument.

When comparing HIV-exposed and HUU infants, the only microbe that appeared to have a distinct presence was that of *Staphylococcus haemolyticus*, one of the most frequent aetiological agents of staphylococcal infections ^40^. The microbe indicated a distinctive high association with HIV exposure at birth and across time points, a finding confirmed by separate statistical tests. *Staphylococcus haemolyticus* has also been commonly found in the hospital setting, with a tendency to become resistant to multiple antibiotics ^41– 43^. Ternes *et al.* (2013) found that 55.9% of infants harbored multidrug-resistant CNS in their nasal cavity, with *S. haemolyticus* being the most frequently isolated species (38.3%) ^44^. However, it should be noted there is a possibility that our genomic identification process could be misidentifying what are actually *S. aureus* reads, which is one of the most common pathogens colonizing the nasopharynx and the lower airways ^45, 46^. *S. aureus* carriage has been found to be significantly higher in RSV than infants infected with rhinovirus ^47^. In a case series from India, NP carriage densities of *Streptococcus pneumoniae* and *S. aureus* were higher in both mothers and children living in HIV-affected households, regardless of the child's HIV status ^48^.

From the stacked barplots, we observed that *Dolosigranulum* seemed to have a higher relative proportion at all time points in HUU infants, but this was not verified as a distinguishable difference in our statistical testing. The microbe *Dolosigranulum pigrum* is generally accepted as a marker of a healthy microbiome, positively associated with *Corynebacterium* and potentially protective against colonization by *S. aureus* and *S. pneumoniae* ^49^. High *Dolosigranulum* carriage has been found to be correlated with positive outcomes of RSV ^50^, COVID-19 ^51^, HIV exposure ^52^, and Bronchiolitis ^53^ as a mediator in defense against illness, albeit the mechanism by which it does so is unclear.

### Associations within mother and infant duad NP microbiome composition over time

When conducting infant-mother duad paired analyses, HIV-exposed infants’ NP microbiome composition was not vastly different from their HIV(+) mothers at birth or 14 weeks, including in their carriage of *S. pneumoniae*, *H. influenzae*, and *S. haemolyticus*. HUU infants were similar to their mothers at birth, but apparently grew apart from their HIV(-) mothers by 14 weeks as infants acquired more *Haemophilus* ( *influenzae*) and decreased in *Staphylococcus haemolyticus* carriage.

### Effect of HIV status on mother NP microbial community composition

One of our study objectives was to compare the NP microbiomes of the HIV(+) and HIV(-) mothers at t=0 and t=6. From Figure 1B, we observed that HIV(+) mothers may have had higher Streptococcus abundance than HIV(-) mothers overall. A significant inter-group beta diversity test result at t=6 showed strong differences in taxa among HIV statuses, a finding reflected by the stacked bar plots indicating a larger presence of taxa with relatively small abundances in HIV(-) women (denoted as “Other” throughout our analyses). This finding implies more microbial diversity in the NP microbiomes of the HIV(-) women. In the nasopharynx, lower diversity has been associated with individuals with rhinovirus illness ^54^ and in children with HIV-associated bronchiectasis ^55^, suggesting that greater NP diversity is a sign of a healthy microbiome. This mirrors previous findings in the gut microbiome of healthy individuals ^56^.

### Study limitations

Based on our results, we see some nuance, but must acknowledge that our study has several limitations. First, and most importantly, given this is both a pilot and a longitudinal study, our sample size is small. Second, given that all samples were collected from participants born in Zambia, these results may not be generalizable to HIV-exposed infants in other countries. Third, as the SAMIPS study was not focused on studying HIV transmission as a main aspect of its structure, several key variables were not measured that would have been informative as potential confounders in our study results. For example, we do not know the viral loads and CD4 counts of the mothers at any point. We also lack information on whether mothers continued to take ART post-pregnancy or were prescribed Bactrim *Pneumocystis jirovecii* Pneumonia (PJP) prophylaxis, which could affect the microbiome. We are also unable to determine whether infants were infected with HIV as proper testing to determine transmission was not conducted as part of the study. Fourth, one infant was noted as having birth asphyxia as a complication experienced during the labor and delivery process ( Table 1). Follow-up questions about severity or treatment were not asked. Given that the study eligibility criteria limited enrollment to healthy infants with no acute or chronic medical conditions, it is unlikely that the birth asphyxia was severe. However, we cannot rule out the use of respiratory instrumentation that could impact the infant’s respiratory microbiome.

These limitations, and especially the small sample sizes, also negatively affect the power of our testing and modeling efforts. This is intended as a pilot study for learning about what trends may be present in this cohort that merit further research with more samples for comparison. It is also of interest as few microbiome studies are longitudinal, providing us with a new perspective on how the NP microbiome may be characterized across time in the different groups.

## Conclusions

Acknowledging these issues, our findings suggest that there are subtle nuances between HIV-exposed and HUU infant populations. The effects we have found here warrant further research and discussion regarding the role HIV exposure plays in infant health before readily affirming or denying that HIV exposure affects infants’ NP microbiomes. The potential effect of the HIV(+) mother’s microbiome on an infant may present further changes in pathogen carriage, or community diversity. If associations hold, identifying HIV exposure as a predisposing factor to illness and poor health in infants would present opportunities for further research and development to support infants’ living situations, especially in areas of higher HIV transmission.

## Consent

Written informed consent for publication of the infants’ patient details was obtained from the parents of the infants. Consent for inclusion of mothers’ data was also obtained from the participating mothers themselves.

## Data Availability

Zenodo: Underlying data for ‘Characterization of longitudinal nasopharyngeal microbiome patterns in maternally HIV-exposed Zambian infants.’ https://doi.org/10.5281/zenodo.7255313 ^25^ This project contains the following underlying data: Data file 1: FinalDatOther.rds Data file 2: FinalDatPICRUSt2.RDS Data file 3: animalculesFinalHIV.rds Data file 4: animalcules_data_2021.rds Data file 5: mappingFaits.csv Data file 6: mappingFinalHIV.csv Data file 7: mappingFinalHIV.tsv Data file 8: preclean_MetaData.txt Data file 9: samips_immunization.csv Data files 10–176: *-sam-report.tsv Data are available under the terms of the Creative Commons Zero "No rights reserved" data waiver (CC0 1.0 Public domain dedication). NCBI BioProject: Characterization of longitudinal nasopharyngeal microbiome patterns in maternally HIV-exposed Zambian infants. Accession number PRJNA874826. https://identifiers.org/NCBI/BioProject:PRJNA874826

## Software availability

Source code available from: https://github.com/aubreyodom/HIV_Exposed_Infants

Archived source code at time of publication: https://doi.org/10.5281/zenodo.7255313 ^25^

License: CC0 1.0 Public domain dedication
