# Supplementary material for: Characterization of longitudinal nasopharyngeal microbiome patterns in maternally HIV-exposed Zambian infants
Source: Gates Open Res. 2024 Aug 30;6:143. Originally published 2022 Nov 11. [Version 2] doi: 10.12688/gatesopenres.14041.2 (PMC11427455; doi:10.12688/gatesopenres.14041.2)
Supplement: Peer review history

# Reviewer report: Reviewer response for version 2

**DOI:** 10.21956/gatesopenres.17682.r37923

**Published:** 2024-09-26

**Authors:** Rupert Kaul

**Affiliations:** 1 University of Toronto, Toronto, Ontario, Canada

Thank you for the opportunity to review the manuscript from Odom and colleagues entitled " Characterization of longitudinal nasopharyngeal microbiome patterns in maternally HIV-exposed Zambian infants". This is a pilot longitudinal study of the nasal microbiota in HIV uninfected and exposed, uninfected Zambian infants. It is an interesting concept, although the small sample size is a major limitation. They find subtle differences and conclude that “further research must be conducted in order to confidently understand the relationship between HIV exposure and infants’ NP microbiomes”.

 1) The rationale is that mortality is increased in HEU infants, with increased rates of pneumonia and diarrhea. There are already several well-defined reasons for this, both based on maternal antibody transfer and socioeconomics. The lack of substantial differences in this pilot study could alternatively have led the authors to conclude that larger nasal microbiome comparisons may not be a fruitful area for large future studies.

 2) The authors state that “Previous studies on the impact of HIV exposure have highlighted strong correlations between HIV exposure and increased risk of pneumococcal colonization and disease7,13,14, whereas others have demonstrated no differences in pathogen carriage between children with HIV infection and control groups15–17. The current analysis seeks to address this important knowledge gap.” However, it does not seem that this study would actually be powered to address this gap.

 3) They also state that “this pilot study could indicate differences encouraging future immunology-focused studies”. Was any thought given to performing some pilot immune studies within the sample set?

 4) It would be useful to provide socioeconomic details of cases and controls, since housing conditions and environment may influence the nasal microbiome and might be expected to vary based on maternal HIV status.

 5) It is unclear why infant HIV testing could not be done to confirm HUU status. Even if this is not part of routine clinical care, PCR testing might be performed if stored blood is available.

 6) “We identified strong dissimilarity between HIV(+) and HIV(-) mothers at t=6 (p <0.01), but higher similarity at t=0 (p = 0.70).” What do the authors hypothesize are the causes for these unexpected variations in maternal microbiome comparability over time?

 7) “The largest difference seemed to occur at t=5. It would seem reasonable to suggest that at birth, infants’ microbiome profiles are more similar than different, and that the diversification of these profiles occurs over time; however, the lack of any considerable difference at t=6 disputes this argument.” It does indeed seem to disprove this argument, which perhaps should therefore be removed?

 8) “…it should be noted there is a possibility that our genomic identification process could be misidentifying what are actually *S. aureus* reads, which is one of the most common pathogens colonizing the nasopharynx”. Since the differences in *S. hemolyticus* are the main difference seen, this seems quite concerning.

Is the work clearly and accurately presented and does it cite the current literature?

Yes

If applicable, is the statistical analysis and its interpretation appropriate?

Partly

Are all the source data underlying the results available to ensure full reproducibility?

Yes

Is the study design appropriate and is the work technically sound?

Partly

Are the conclusions drawn adequately supported by the results?

Partly

Are sufficient details of methods and analysis provided to allow replication by others?

Yes

Reviewer Expertise:

Genital microbiome, genital immunology, HIV transmission.

I confirm that I have read this submission and believe that I have an appropriate level of expertise to confirm that it is of an acceptable scientific standard, however I have significant reservations, as outlined above.

# Reviewer report: Reviewer response for version 2

**DOI:** 10.21956/gatesopenres.17682.r37848

**Published:** 2024-09-24

**Authors:** Bryan Vonasek

**Affiliations:** 1 Michigan State University College of Osteopathic Medicine (Ringgold ID: 43977), East Lansing, Michigan, USA

All of my previous comments have been adequately addressed.

Is the work clearly and accurately presented and does it cite the current literature?

Partly

If applicable, is the statistical analysis and its interpretation appropriate?

Partly

Are all the source data underlying the results available to ensure full reproducibility?

Yes

Is the study design appropriate and is the work technically sound?

Partly

Are the conclusions drawn adequately supported by the results?

No

Are sufficient details of methods and analysis provided to allow replication by others?

Yes

Reviewer Expertise:

Pediatric infectious diseases

I confirm that I have read this submission and believe that I have an appropriate level of expertise to confirm that it is of an acceptable scientific standard.

# Reviewer report: Reviewer response for version 1

**DOI:** 10.21956/gatesopenres.15340.r36374

**Published:** 2024-07-12

**Authors:** Bryan Vonasek

**Affiliations:** 1 Michigan State University College of Osteopathic Medicine (Ringgold ID: 43977), East Lansing, Michigan, USA

This is an interesting study that investigates an important question: why do HIV exposed infants have poor health outcomes compared to unexposed infants? The key aspects of this study that are more novel are 1) longitudinal analysis of the microbiome and 2) focus on the NP microbiome. Especially when combined with the paired microbiome data from the mothers and the nice use of controls (HUU infants), this is a good study design that can be scaled up from this exploratory/pilot study. 

 As the authors do a good job of describing and acknowledging, it's hard to glean much from these data given the small sample sizes. Especially considering the wide variation within groups that is described, making comparisons between groups then becomes challenging. That said, I think it is important to  Index these findings, but I would first like to see several improvements to allow readers to better understand: 1) the results, 2) some additional key limitations, and 3) coherent interpretations of the results.

General considerations

 1. The authors acknowledge the limitation of not actually knowing whether the exposed infants are uninfected. I would be extra-careful with this consideration. Don't define them as HEU, maybe instead just "HIV exposed."

 2. From what I can tell, a P-value cutoff to define statistically significant differences was not defined in the Methods. Of course, <0.05 is common and seems to be used in the bolding of some of the P-values in the Results. Especially for a study like this in which there are so many potential confounders that aren't really being addressed and in which the sample size is limited, I would be really careful going too much into differences that aren't statistically significant. Phrases throughout the Results and Discussion like "marginally significant" and "nominally significant" should be de-emphasized or removed. Especially in the Discussion, focus on the significant differences that you did find (including a coherent discussion about these differences), rather than emphasizing too much about differences that weren't significant.

 3. The finding of more Staph haemolyticus in the exposed babies at birth is certainly worth highlighting (even more than you already do). But it's confusing as written in the Abstract and in the text: "...even when accounting for the interaction between HIV exposure status and time of sampling." Why "account for" exposure when that is the input variable you're looking at? Why "account for" time of sampling when they are all "at birth." This can be worded more clearly. Also, know that Staph haemolyticus can certainly be pathogenic in humans, especially in immunocompromised individuals (i.e. newborns, HIV exposed or infected)--you can revamp the Discussion with this in mind. In relation to this, also worth commenting on whether the HIV(+) mothers were more likely or not to have S. haemolyticus. 

 4. A big factor not addressed is whether the exposed infants and their mothers living with HIV were on Bactrim prophylaxis. If you don't know, this needs to be clearly stated as a limitation. If you do know, please incorporate as a covariate in your analyses. Bactrim would likely have a major impact on the NP microbiome.

 5. The manuscript will be much more digestible for the reader describe the various analyses you are doing here in a parallel order in the Methods, Results, and Discussion.

Introduction

 1. Overall, this is well written. In fact, the quality of the Introduction compared to the Discussion is so striking to me that I suspect different authors being mainly responsible for these two sections? If I'm correct, then the author that led writing of the Intro should please help polish up the Discussion!

 2. "We collected 167 samples...through 14 weeks of life." >> this sentence doesn't belong in the Introduction.

Methods

 1. "Characteristics of the study cohort are in Table 1." >> this goes in the Results.

 2. Important to give some sense of how the 10 exposed infants were sampled for inclusion in this study from the larger study. Its says it was those entering the study from April to June--all of them from the parent study? Or some sub-set? Random selection?

 3. I also think the text related to Table 2 would be more appropriate in the Results.

Results

 1. Table 1 -- Remove the rows without any data (Chilenje clinic, father lives with child, all rows are trimester starting HIV)

 2. First paragraph: Ages of infants at time of swabbing doesn't match what I see in Table 2. Along these lines, be consistent using either days or weeks.

 3. Second paragraph: "...which may in part be the result of..." >> interpretation of data belongs in the Discussion.

 4. Fig 1. "120 days" -- This is different than 14 weeks?

 5. Third paragraph: Rephrase the first sentence because "heavy presence" and "relatively low" are contradictory. Maybe "diversity" instead of "presence"?

 6. "These transitional patterns align with..." >> this belongs in the Discussion.

 7. Paragraph starting with "While longitudinal trends..." To me, this should be the first paragraph under the existing heading "Relationship between NP microbiome comp...".

 8. The paragraph and data in the table about the Teo study are rather distracting. At the very least, all this needs to be moved to the Discussion. That cohort is very different from the cohort in this study, so I suggest not allocating so much text to that--maybe just a brief comparison as you do with ref 32 (which, again, belongs in the Discussion).

 9. Fig 2 -- Define the widths of the lines plotted in the figure description. Double check, and consider rephrasing, your description of the slopes: I'm not sure what "accounted for" means.

 10. "As previously noted, we chose p=6..." >> No need to reiterate.

 11. "...difficulty in identifying clear differences between populations." >> rephrase (even remove!) as this reads as if you're assuming that there are differences.

 12. "Overall microbiome composition varied significantly..." >> Isn't this redundant with what is written at the beginning of the Results? 

 13. The paragraph describing beta-diversity needs to be revamped and written more clearly.

 14. In the tables and text, just use one of these terms and be consistent throughout: "HEU/HIV(+), etc." vs "HIV(+)/HEU" -- flipping the order around confuses the reader.

 15. The sentences about Strep pneumo differences are confusing. Reword to mirror how H. influenzae is described--though in that sentence, write "more abundant" not "more highly abundant." Write your sentence re S. haemolyticus in the same way.

 16. Also be consistent with how you write about the time points: "t=6" vs "time point six" vs "last time point" >> just pick one way and stick with it.

 17. Table 7 -- Reorder the columns to a more conventional order: Mean, 95% CIs, P-value (left to right). Consider removing 't' and 'df' columns. In the table heading, mention what variables you are adjusting for to get the "adjusted p-value." Why are you now using "HIV" and "Control" to define the groups? I suggest naming the groups the same as for Table 6, unless you have a good reason.

Discussion

 Overall, this needs a lot of improvement. 

 1. The first paragraph should summarize the keys findings from YOUR data.

 2. In the second paragraph, maybe just clearly state microbiome as a third possible hypotheses and acknowledge that all three drivers are likely inter-related and hard to differentiate.

 3. Be clear when describing differences in the NP microbiome in the exposed infants 1) longitudinally vs 2) compared to un-exposed infants vs 3) c/t mothers. This is an multiple areas. I can certainly infer your meaning, but it would be easier to read if the comparison is clearly reiterated.

 4. The connection between description of Dolosigranulum and the cited studies is not clear. 

 5. Rather than describing the diversity of the microbiome for the mothers as having lots of "other" organisms, wouldn't it be more valid to look formally at beta and alpha diversity?

 6. You argue pretty clearly that you have good samples. I don't think you need to state "retrospective analysis of samples" as a limitation. 

 7. I don't agree with the statements about minimizing the limitation of lack of HIV testing. In this age group, HIV infections can certainly be rapidly progressive. Also, because the underlying causes of HEU having poor health c/t HUU aren't well understood in the first place, one can't assume the effects ("direction of bias") of contaminating the HEU cohort with infected infants.   

 8. "This is intended as a case study..." >> Use the term "exploratory" or "pilot" study instead because "case study" has a different, specific meaning.

Conclusions

 Focus here on what can be gleaned from your study results. You did not evaluate "sociological differences."

Consent

 Because you also report data from the mothers, I hope you also consented them for that (not just the infant's data)?

Is the work clearly and accurately presented and does it cite the current literature?

Partly

If applicable, is the statistical analysis and its interpretation appropriate?

Partly

Are all the source data underlying the results available to ensure full reproducibility?

Yes

Is the study design appropriate and is the work technically sound?

Partly

Are the conclusions drawn adequately supported by the results?

No

Are sufficient details of methods and analysis provided to allow replication by others?

Yes

Reviewer Expertise:

Pediatric infectious diseases

I confirm that I have read this submission and believe that I have an appropriate level of expertise to confirm that it is of an acceptable scientific standard, however I have significant reservations, as outlined above.

# Response

**Published:** 2024-08-13

**Authors:** Aubrey Odom-Mabey

**Affiliations:** Boston University Medical Campus, USA

Reviewer 2

Comment 2-1: This is an interesting study that investigates an important question: why do HIV exposed infants have poor health outcomes compared to unexposed infants? The key aspects of this study that are more novel are 1) longitudinal analysis of the microbiome and 2) focus on the NP microbiome. Especially when combined with the paired microbiome data from the mothers and the nice use of controls (HUU infants), this is a good study design that can be scaled up from this exploratory/pilot study. 

 As the authors do a good job of describing and acknowledging, it's hard to glean much from these data given the small sample sizes. Especially considering the wide variation within groups that is described, making comparisons between groups then becomes challenging. That said, I think it is important to  Index these findings, but I would first like to see several improvements to allow readers to better understand: 1) the results, 2) some additional key limitations, and 3) coherent interpretations of the results.

Response 2-1: We thank the reviewer for providing thorough, thoughtful feedback for this paper.

Comment 2-2: The authors acknowledge the limitation of not actually knowing whether the exposed infants are uninfected. I would be extra-careful with this consideration. Don't define them as HEU, maybe instead just "HIV exposed."

Response 2-2: We thank the reviewer for this feedback. We have changed all instances of “HEU” throughout the manuscript to “HIV-exposed”.

Comment 2-3: From what I can tell, a P-value cutoff to define statistically significant differences was not defined in the Methods. Of course, <0.05 is common and seems to be used in the bolding of some of the P-values in the Results. Especially for a study like this in which there are so many potential confounders that aren't really being addressed and in which the sample size is limited, I would be really careful going too much into differences that aren't statistically significant. Phrases throughout the Results and Discussion like "marginally significant" and "nominally significant" should be de-emphasized or removed. Especially in the Discussion, focus on the significant differences that you did find (including a coherent discussion about these differences), rather than emphasizing too much about differences that weren't significant.

Response 2-3: We have clarified in the methods that α=0.05 (see line 216). Where relevant, a Bonferroni correction is applied to adjust the alpha value (type I error rate) to account for Type I error rate inflation as noted for the GEE and the Hotelling’s T-squared test methods descriptions, and elsewhere in the manuscript (e.g. the table descriptions).

 Our rationale for including “marginally significant” results is that Bonferroni corrections are known to be at times overly conservative in deflating Type I error, and thus adjusted p-values that are around the newly calculated alpha value can still be practically interesting. To make this more clear for the reader, we added the following sentence to the methods section:

 “Due to the conservative nature of the Bonferroni correction, adjusted p-values between 0.05 and 0.10 were noted as marginally significant as effects that could retain some level of practical significance.”

 We removed a few results that were greater than the cutoff of 0.10 and altered the discussion accordingly.

Comment 2-4: The finding of more *Staph haemolyticus* in the exposed babies at birth is certainly worth highlighting (even more than you already do). But it's confusing as written in the Abstract and in the text: "...even when accounting for the interaction between HIV exposure status and time of sampling." Why "account for" exposure when that is the input variable you're looking at? Why "account for" time of sampling when they are all "at birth." This can be worded more clearly.

 Also, know that Staph haemolyticus can certainly be pathogenic in humans, especially in immunocompromised individuals (i.e. newborns, HIV exposed or infected)--you can revamp the Discussion with this in mind. In relation to this, also worth commenting on whether the HIV(+) mothers were more likely or not to have S. haemolyticus.

Response 2-4: We can see how this wording can be confusing to the reader. The inclusion of a variable in a statistical model is by definition to acknowledge or account for the quantifiable effect of that variable on the response. The wording in the abstract is meant to convey that concept. We can understand how this might not be clear to readers lacking a statistical background, and so we rewrote these points:

 [Discussion] The microbe indicated a distinctive high association with HIV exposure at birth and across time points, a finding confirmed by separate statistical tests. (lines 452-453)

 [Abstract] When evaluating the interaction between HIV exposure status and time of sampling among infants, the microbe *Staphylococcus haemolyticus* showed a distinctive high association with HIV exposure at birth. (lines 29-31)

Comment 2-5: A big factor not addressed is whether the exposed infants and their mothers living with HIV were on Bactrim prophylaxis. If you don't know, this needs to be clearly stated as a limitation. If you do know, please incorporate as a covariate in your analyses. Bactrim would likely have a major impact on the NP microbiome.

Response 2-5: We thank the reviewer for this observation. Unfortunately, the SAMIPS study did not track Bactrim Pneumocystis jirovecii Pneumonia (PJP) prophylaxis, only acute use of antibiotics in response to infection. This cohort required that infants were healthy as a criterion of limitation, and so antibiotic use in response to infection was not included as a covariate.

 We have acknowledged this limitation in the discussion section (lines 494-496).

Comment 2-6: The manuscript will be much more digestible for the reader describe the various analyses you are doing here in a parallel order in the Methods, Results, and Discussion.

Response 2-6: We agree that the current delineation of analyses is difficult to follow, and have implemented the following changes throughout the Methods, Results, and Discussion, with an emphasis on reorganizing text under appropriately titled, standardized subheadings.

 Changes to subheadings themselves were made as follows.

- In the methods section, we replaced the “Statistical analysis” subheading with ones that referenced the type of analysis conducted. The wording was reflected in the Results and Discussion subsections. These are: “Visualizing longitudinal trends in microbiome composition”, “Modeling effects of HIV status on infant microbiome composition across time”, and “Testing differences in microbiome composition across time and cohort groups”. These subheadings are broad enough to allow all analyses to be grouped together more concisely by method.
- In the Results section, we made the following changes to the previous subheadings:
  - “Longitudinal differences in the nasopharyngeal microbiome composition” was rewritten as “Visualizing longitudinal trends in NP microbiome composition”
  - “Relationship between nasopharyngeal microbiome composition and HIV exposure” was rewritten as “Effects of HIV status on infant NP microbiome composition across time”
  - “Relationship between infant and mother nasopharyngeal microbiome composition across time” was rewritten as “Associations within mother and infant duad NP microbiome composition over time”
  - “Trends in NP microbial community composition between HIV(+) and HIV(-) mothers” was rewritten as “Effect of HIV status on mother NP microbial community composition”
- In the Discussion, we added subheaders that were nearly identical to the Results subheaders.

Introduction

Comment 2-7: Overall, this is well written. In fact, the quality of the Introduction compared to the Discussion is so striking to me that I suspect different authors being mainly responsible for these two sections? If I'm correct, then the author that led writing of the Intro should please help polish up the Discussion!

Response 2-7: We have updated the wording in the Discussion based on suggestions provided by each reviewer, including the order of results discussed, the relocation of two paragraphs discussing previous findings in the literature, and the addition of literature examining *S. haemolyticus.*

Comment 2-8: "We collected 167 samples...through 14 weeks of life." >> this sentence doesn't belong in the Introduction.

Response 2-8: We have rewritten the relevant sentences of the introduction to remove specific mentions of sample numbers or exact time of analyses to focus on the context of the study (i.e., the locale, the infant groups, and the NP microbiome sampling). This now reads as follows:

 “Given the complex dynamics of interactions between the host, microbes, and environment beginning at birth, we conducted an exploratory longitudinal comparison of the nasopharyngeal (NP) microbiomes of HEU and HUU infants and their mothers during the first 14 weeks of life. We reasoned that by characterizing quantifiable differences in NP microbiota distinguishing these two groups, this pilot study could indicate differences encouraging future immunology-focused studies” (lines 81-83).

Methods

Comment 2-9: "Characteristics of the study cohort are in Table 1." >> this goes in the Results.

Response 2-9: We have moved Table 1 and some accompanying text to the Results section (line 267).

Comment 2-10: Important to give some sense of how the 10 exposed infants were sampled for inclusion in this study from the larger study. Its says it was those entering the study from April to June--all of them from the parent study? Or some sub-set? Random selection?

Response 2-10: We added the following clarification to the first paragraph of the Methods section (see lines 89-94):

 From 1,981 total mother infant pairs, we selected a subset that had 3 or more study visits, had no siblings under the age of five years, and who enrolled in the study from April to July 2015.  From this subset, we randomly selected 10 mother infant pairs with an HIV positive mother who started ARV treatment before pregnancy.  These 10 mother infant pairs were randomly matched to 10 mother infant pairs with an HIV negative mother by education, month of entry into the study, and maternal age.

Comment 2-11: I also think the text related to Table 2 would be more appropriate in the Results.

Response 2-11: We have moved Table 2 and some accompanying text to the Results section (line 268-273).

Results

Comment 2-12: First paragraph: Ages of infants at time of swabbing doesn't match what I see in Table 2. Along these lines, be consistent using either days or weeks.

Response 2-12: We note the discrepancy and have removed the delineation of days in the first column of Table 2. We updated Table 1 to reflect weeks at enrollment rather than days. We changed the delineation of days at swabbing time points to weeks.

Comment 2-13: Table 1 -- Remove the rows without any data (Chilenje clinic, father lives with child, all rows are trimester starting HIV)

Response 2-13: These rows have been removed and Table 1 has been restructured to better delineate parameter sections/groups.

Comment 2-14: Second paragraph: "...which may in part be the result of..." >> interpretation of data belongs in the Discussion.

Response 2-14: The paragraph on the Teo et al. study has been moved to the discussion section.

Comment 2-15: Fig 1. "120 days" -- This is different than 14 weeks?

Response 2-15: We believe this was written in error. We have changed this caption to read “14 weeks”.

Comment 2-16: Third paragraph: Rephrase the first sentence because "heavy presence" and "relatively low" are contradictory. Maybe "diversity" instead of "presence"?

Response 2-16: We have altered this sentence to read “Due to the high diversity of taxa with relatively low abundance among individual microbes…” (line 288-289).

Comment 2-17: "These transitional patterns align with..." >> this belongs in the Discussion.

Response 2-17: This sentence has been relocated to the discussion section (lines 423-433).

Comment 2-18: Paragraph starting with "While longitudinal trends..." To me, this should be the first paragraph under the existing heading "Relationship between NP microbiome comp...".

Response 2-18: We agree with the reviewer and have moved that paragraph to the heading titled “Relationship between nasopharyngeal microbiome composition and HIV exposure.” (lines 312-316)

Comment 2-19: The paragraph and data in the table about the Teo study are rather distracting. At the very least, all this needs to be moved to the Discussion. That cohort is very different from the cohort in this study, so I suggest not allocating so much text to that--maybe just a brief comparison as you do with ref 32 (which, again, belongs in the Discussion).

Response 2-19: We relocated the Teo et al. study to the discussion and renumbered the tables accordingly (lines 423-433). The text discussing the study has been shortened to remove some excess discussion of differences while still introducing the cohort and thus retaining the table’s relevance.

Comment 2-20: Fig 2 -- Define the widths of the lines plotted in the figure description. Double check, and consider rephrasing, your description of the slopes: I'm not sure what "accounted for" means.

Response 2-20: We agree with the reviewer that the explanation of the figure according to the caption is difficult to parse, and so we have rewritten the caption for clarity in delineating each visual aspect of the plot grid. We removed the “accounted for” phrasing.

 “Plots of estimated marginal means of the abundance of a given microbe, given the estimated GEE effects of time point of sampling (x axis) and HIV status (line color). A line illustrates the estimated change in microbe abundance over time, with positive or negative slopes illustrating increased or decreased estimated abundance in log CPM (respectively). HIV-exposed infants (denoted as HEU) are depicted in blue, and HIV-unexposed, uninfected infants (HUU) are depicted in red. Given the separate lines for each status and changing slope across time, these plots depict the interaction effect between time point and HIV status. All microbes had at least marginally significant time effects, but only S. haemolyticus had a very strong HIV exposure status effect.”

Comment 2-21: "As previously noted, we chose p=6..." >> No need to reiterate.

Response 2-21: We have removed this sentence from the Results section.

Comment 2-22: "...difficulty in identifying clear differences between populations." >> rephrase (even remove!) as this reads as if you're assuming that there are differences.

Response 2-22: We agree with the reviewer and acknowledge that our point may have been difficult to grasp especially for audiences without familiarity with the test in question. We have removed the sentence from the paragraph to avoid confusion.

Comment 2-23: "Overall microbiome composition varied significantly..." >> Isn't this redundant with what is written at the beginning of the Results? 

Response 2-23: We have reread the Results and it is unclear what section the reviewer is referring to as redundant. We have retained this section as is.

Comment 2-24: The paragraph describing beta-diversity needs to be revamped and written more clearly.

Response 2-24: We rewrote the beta diversity paragraph describing infant results as follows:

 “We examined variations in overall microbiome composition by observing differences in beta diversity between HUU and HIV-exposed infants. We conducted two Wilcoxon rank-sum tests of the Bray-Curtis dissimilarity at t=0 and t=6. We found a non-significant difference in microbiome composition between HUU and HIV-exposed infants at t=0 (p = 0.68), but large differences at t=6 (p <0.01). Performing additional tests within the HIV-exposed and HUU groups themselves, we also identified heavy inter-subject variability at both t=0 and t=6 (all p-values < 0.01). As a result of this inter-subject variability, alpha diversity metrics were not performed for this analysis.” (lines 339-345)

Comment 2-25: In the tables and text, just use one of these terms and be consistent throughout: "HEU/HIV(+), etc." vs "HIV(+)/HEU" -- flipping the order around confuses the reader.

Response 2-25: We have replaced all instances of status groups with HEU/HIV(+) and HUU/HIV(-), and replaced “mother-infant” with “infant-mother” pairs to match the ordering of the descriptors.

Comment 2-26: The sentences about Strep pneumo differences are confusing. Reword to mirror how H. influenzae is described--though in that sentence, write "more abundant" not "more highly abundant." Write your sentence re S. haemolyticus in the same way.

Response 2-26: We rewrote the test results as follows (lines 369-372):

*“H. influenzae* was more abundant in HUU infants than in their HIV(-) mothers at *t = 6* (adj. p = 0.1), and *S. haemolyticus* was likely to be found in the HUU infants at t = 0 in a higher concentration than in their HIV(-) mothers (adj. p = 0.1). *S. pneumoniae *was more likely to be found in HUU infants than their HIV(-) mothers at *t *= 6 (adj. p = 0.1). Each of these findings were marginally significant.”

Comment 2-27: Also be consistent with how you write about the time points: "t=6" vs "time point six" vs "last time point" >> just pick one way and stick with it.

Response 2-27: We thank the reviewer for this feedback. We decided to change all instances of alternate references to time points to variable notation (e.g., t=0 or t=6).

Comment 2-28: Table 7 -- Reorder the columns to a more conventional order: Mean, 95% CIs, P-value (left to right). Consider removing 't' and 'df' columns. In the table heading, mention what variables you are adjusting for to get the "adjusted p-value." Why are you now using "HIV" and "Control" to define the groups? I suggest naming the groups the same as for Table 6, unless you have a good reason.

Response 2-28: We have reordered the columns to reflect the suggested order and removed the t statistic and degrees of freedom columns to reduce table information. We also relabeled “HIV” and “Control” to HEU/HIV(+) and HUU/HIV(-), which reflects the Table 6 nomenclature. We noted in the table caption that a Bonferroni correction was used to obtain the adjusted p-values.

 Table 7 was renamed as Table 6.

Discussion

Comment 2-29: The first paragraph should summarize the keys findings from YOUR data.

Response 2-29: We moved the paragraph discussing previous findings in the literature to after the initial paragraph (line 400). We combined the first two paragraphs to now compose the first paragraph of the discussion, which gives an overview of our findings (that there is nuance).

Comment 2-30: In the second paragraph, maybe just clearly state microbiome as a third possible hypotheses and acknowledge that all three drivers are likely inter-related and hard to differentiate.

Response 2-30: We thank the reviewer for this feedback. Given that the hypotheses we proposed were related to microbiota differences as potential upstream or downstream indicators, we have adjusted our wording to read as follows:

 “Assuming that such differences are not merely due to sampling biases, the chief hypotheses explaining them are that 1) HEU infants have subtle immunological deficits, or 2) these are the consequence of confounding due to environmental and sociological factors. These are not mutually exclusive, and we acknowledge that all drivers are likely inter-related and difficult to differentiate. We reasoned that they may be in turn associated with differences in the microbiota which could then serve as a quantifiable indicator of differences in respiratory health between HEU and HUU infants.” (lines 392-394)

Comment 2-31: Be clear when describing differences in the NP microbiome in the exposed infants 1) longitudinally vs 2) compared to un-exposed infants vs 3) c/t mothers. This is an multiple areas. I can certainly infer your meaning, but it would be easier to read if the comparison is clearly reiterated.

Response 2-31: We adjusted unclear wording and added headers throughout the discussion to properly delineate the various analysis types (see Response 2-6).

Comment 2-32: The connection between description of Dolosigranulum and the cited studies is not clear. 

Response 2-32: We have rewritten the paragraph discussing *Dolosigranulum* in the results section (lines 463-469) to better describe *Dolosigranulum pigrum *in the literature but refrain from making conclusions based on the data given the lack of statistically significant results.

Comment 2-33: Rather than describing the diversity of the microbiome for the mothers as having lots of "other" organisms, wouldn't it be more valid to look formally at beta and alpha diversity?

Response 2-33: It appears that the interpretation of beta diversity results was only implied at the time of writing, and so we have rewritten this paragraph to explain the results more clearly:

 “One of our study objectives was to compare the NP microbiomes of the HIV(+) and HIV(-) mothers at t=0 and t=6. From Figure 1B, we observed that HIV(+) mothers may have had higher Streptococcus abundance than HIV(-) mothers overall. A significant inter-group beta diversity test result at t=6 showed strong differences in taxa among HIV statuses, a finding reflected by the stacked bar plots indicating a larger presence of taxa with relatively small abundances in HIV(-) women (denoted as “Other” throughout our analyses). This finding implies more microbial diversity in the NP microbiomes of the HIV(-) women. In the nasopharynx, lower diversity has been associated with individuals with rhinovirus illness50 and in children with HIV-associated bronchiectasis,51 suggesting that greater NP diversity is a sign of a healthy microbiome. This mirrors previous findings in the gut microbiome of healthy individuals.52” (lines 477-486)

Comment 2-34: You argue pretty clearly that you have good samples. I don't think you need to state "retrospective analysis of samples" as a limitation. 

Response 2-34: We agree with the reviewer and have removed this limitation from the Conclusion section.

Comment 2-35: I don't agree with the statements about minimizing the limitation of lack of HIV testing. In this age group, HIV infections can certainly be rapidly progressive. Also, because the underlying causes of HEU having poor health c/t HUU aren't well understood in the first place, one can't assume the effects ("direction of bias") of contaminating the HEU cohort with infected infants.   

Response 2-35: We have removed the two sentences in question that served to minimize the argument against HIV incidence in the cohort.

Comment 2-36: "This is intended as a case study..." >> Use the term "exploratory" or "pilot" study instead because "case study" has a different, specific meaning.

Response 2-36: We have corrected this oversight by using the term “pilot study” (line 504).

Conclusions

Comment 2-37: Focus here on what can be gleaned from your study results. You did not evaluate "sociological differences."

Response 2-37: We appreciate the reviewer’s remarks and have subsequently removed references to sociological differences in the Conclusion. We have altered the first sentence to read as follows:

 “Acknowledging these issues, our findings suggest that there are subtle nuances between HIV-exposed and HUU infant populations.” (lines 509-510)

Consent

Comment 2-38: Because you also report data from the mothers, I hope you also consented them for that (not just the infant's data)?

Response 2-38: We thank the reviewer for noticing this oversight. We did obtain consent from the mothers who were also participants in the study. We have added the following to the Consent section of the manuscript (lines 554-556):

 “Written informed consent for publication of the infants’ patient details was obtained from the parents of the infants. Consent for inclusion of mothers’ data was also obtained from the participating mothers themselves.”

# Reviewer report: Reviewer response for version 1

**DOI:** 10.21956/gatesopenres.15340.r33429

**Published:** 2023-06-16

**Authors:** Christiana Smith

**Affiliations:** 1 University of Colorado, Aurora, CO, USA

This is a well-written description of a very small study comparing the nasopharyngeal microbiome of Zambian HEU infants (and their mothers) to HUU infants (and their mothers) over the first 14 weeks of life. The hypothesis that the maternal or infant microbiome plays a key role in the increased morbidity/mortality observed in HEU infants has been under-explored in the literature, justifying the indication for the study. However, the differences identified between HEU and HUU infants in this study were rather minimal (the authors use the word “subtle”), which diminishes the overall impact/significance of the publication.

 Strengths of the study include that the participants were followed longitudinally over time, and the HEU and HUU cohorts were well-matched and recruited from the same physical location.

 The two major limitations of the study, which are described appropriately by the authors, are: 1) the small sample size results in very limited power to identify differences between HEU and HUU infants, leading to “uncertain evidence that there is an HIV exposure effect on the NP microbiome...” and 2) no information is provided on the immune status of the HIV+ mothers (viral load, CD4) or the HIV infection status of the HIV-exposed infants (i.e. HIV NAAT results after birth), which are likely very important contributors to the NP microbiome. For example – a person with poorly controlled HIV and low CD4 count is at risk of opportunistic respiratory infections (TB, PJP, MAC) which would likely influence the composition of their NP microbiome. Was there really no information available to the study authors that could be included to better describe the health of the mothers and infants (any maternal viral loads or CD4 counts, known maternal comorbidities, infant dried blood spot testing at birth, etc.)?

 Person-first language should be used throughout the abstract and manuscript. It is probably fine to use the abbreviations as-is (HIV+, HEU, HUU), but phrases such as “HIV-positive mothers” or “HIV-infected women” should be changed to “women/mothers with HIV” or “women/mothers living with HIV.” The term “mother-to-child transmission” is also outdated and should be changed to “vertical transmission” or “perinatal transmission.” For more information on why this matters, please see: Dilmitis *et al.* (2012 ^1^).

 Introduction, paragraph 2: the authors reference “the HEU phenomenon” several times without really explaining what “the phenomenon” is. Are they referring to the increased morbidity and mortality observed in this population? Please be more specific.

 Introduction, paragraph 4: The authors state that their study might “provide insight into the presence or absence of immunological factors distinguishing [HEU vs. HUU infants].” However, differences in the microbiome do not necessarily indicate differences in “immunological factors.” Recommend revising the text appropriately.

 Methods, paragraph 1: it is necessary to state whether all infants breastfed or whether some infants consumed any foods other than breast milk in the first 14 weeks of life, as nutrition sources have a profound ability to impact the microbiome.

 There are several examples where the manuscript is organized in a non-standard way:

- Tables containing data (such as Table 1 and Table 2) belong in the results section, not the methods section. Recommend moving these tables, and the text describing them, to the beginning of Results.
- The text in the methods section describing the reasons that some samples were excluded (beginning with “Some missingness in the data is present…”) also belongs in results, not methods.
- There are some “editorial” comments in the results section, which really belong in the discussion instead. For example, page 7, last paragraph: “…which may in part be the result of the mothers’ acquired immunity over time and therefore lower overall NP carriage;” page 8, 3 ^rd^ to last paragraph: “These transitional patterns align with those found in a longitudinal East Asian infant cohort.”

Table 1 is very confusing and poorly organized:

- The headers often do not apply to the rows beneath them (male sex and median age should not be categorized under “place of birth;” median birth weight and twin births should not be categorized under “gestational age;” number of mothers, mothers age, married, father lives with child should not be categorized under “immunizations at birth;” etc.
- There are many rows for which the result is 0 in every column, or for which there are apparently no data. These rows should be removed from the table completely. For example, why is the Chilenje clinic described in table when only the Chawama clinic is described in the text? Why is “other place of birth” listed in the table if none of the infants were born in an “other place”? Why are “father lives with child” and “trimester initiated ART” included in the table if there are no data for these variables? The L&D complications that did not occur in any infants (obstructed labor, sepsis, hemorrhage, other) can be removed and a sentence could be added to the text that says “none of these complications occurred: X,Y,Z.”
- The table shows one infant with birth asphyxia. This infant should be further described in the text. How severe was the birth asphyxia? Did the infant require respiratory support, intubation, oxygen, etc. (since respiratory instrumentation could potentially have impacted this infant’s respiratory microbiome)?

Table 2:

- The text states that two of 20 mothers lacked swabs at both the t=0 and t=6 time points. However, the table shows all 20 mothers had a swab at t=0 and 2 were missing swabs at t=6. Either the text or table should be revised so that they harmonize.
- It would be preferred if the table could be stratified by HIV status at each time point, rather than consolidating all of the HEU and HUU results at the bottom. This way it would be easy to see whether either group was over-represented at any one given time point.

Table 3: please standardize the number of decimal points across columns (some columns have 2 decimal points, others have none).

 Table 4: several columns describe “adjusted p-values” but there is no description of what variables were adjusted for. The reader should not have to refer back to the methods section to understand the statistical analysis. This is also a problem in Table 7. Please add a description of what variables the analyses were adjusted for to the Table heading or to a footnote for each table.

 Table 6: I believe there is a typo in the table; the two time points being compared are t=0 and t=6 according to the text; however, the table shows t=0 and t=1.

 Table 7: It is very difficult to understand this table without reading the text. Specifically, what direction is indicated by the positive and negative numbers in the column titled “mean of differences” (higher abundance in infants or higher abundance in mothers)? After a long period of scrutiny, I was able to determine that negative numbers mean higher abundance in infants, but it should be immediately apparent to the reader. Please explain this in the Table heading or in a footnote.

 Also related to Table 7, in the results section, page 12, paragraph 2: The authors describe that the purpose of the analysis in Table 7 is to compare the abundance of 3 particular bacterial species between the mothers and infants within each cohort (HIV+/HEU and HIV-/HUU), and that this comparison will help explain why HEU infants may be more susceptible to morbidity/mortality. It is not clear how comparing the abundance of these bacteria between mothers vs. infants WITHIN each cohort would help explain the HEU infants’ increased susceptibility to morbidity/mortality. It seems that to answer this question, it would be more valuable to compare the abundance of bacteria between HEU vs. HUU infants (or between HIV+ vs. HIV- mothers). Please clarify how you expect this analysis to answer the question proposed, or modify/remove the language describing the reason for this analysis.

 Discussion: The authors should add to the list of limitations that their results may not be generalizable to HEU infants around the globe, given that all samples were collected from participants living in a single African country.

 Discussion, page 14: There is some redundancy between the last 2 sentences of the first paragraph, and the 6 ^th^ paragraph, both of which describe differences in bacterial diversity between HIV+ and HIV- mothers.

 There are several typos in the manuscript. For example, page 11, paragraph 3: “The same test for all infant samples at time point six was contrastingly showed….” Also Conclusions: “our findings may suggest that that sociological….”

Is the work clearly and accurately presented and does it cite the current literature?

Yes

If applicable, is the statistical analysis and its interpretation appropriate?

I cannot comment. A qualified statistician is required.

Are all the source data underlying the results available to ensure full reproducibility?

Yes

Is the study design appropriate and is the work technically sound?

Yes

Are the conclusions drawn adequately supported by the results?

Yes

Are sufficient details of methods and analysis provided to allow replication by others?

Yes

Reviewer Expertise:

Pediatric infectious diseases clinician, research focused on the immunology of HIV-exposed and HIV-infected infants and children.

I confirm that I have read this submission and believe that I have an appropriate level of expertise to confirm that it is of an acceptable scientific standard, however I have significant reservations, as outlined above.

## References

[1] : Language, identity and HIV: why do we keep talking about the responsible and responsive use of language? Language matters. *Journal of the International AIDS Society* .2012;15(4(Suppl 2)) : 10.7448/IAS.15.4.17990 10.7448/IAS.15.4.17990 22360788

# Response

**Published:** 2024-08-13

**Authors:** Aubrey Odom-Mabey

**Affiliations:** Boston University Medical Campus, USA

Reviewer 1

Comment 1-1: This is a well-written description of a very small study comparing the nasopharyngeal microbiome of Zambian HEU infants (and their mothers) to HUU infants (and their mothers) over the first 14 weeks of life. The hypothesis that the maternal or infant microbiome plays a key role in the increased morbidity/mortality observed in HEU infants has been under-explored in the literature, justifying the indication for the study. However, the differences identified between HEU and HUU infants in this study were rather minimal (the authors use the word “subtle”), which diminishes the overall impact/significance of the publication.

 Strengths of the study include that the participants were followed longitudinally over time, and the HEU and HUU cohorts were well-matched and recruited from the same physical location.

Response 1-1: We appreciate the reviewer’s summary and thank the reviewer for providing their feedback for this paper.

Comment 1-2: The two major limitations of the study, which are described appropriately by the authors, are: 1) the small sample size results in very limited power to identify differences between HEU and HUU infants, leading to “uncertain evidence that there is an HIV exposure effect on the NP microbiome...” and 2) no information is provided on the immune status of the HIV+ mothers (viral load, CD4) or the HIV infection status of the HIV-exposed infants (i.e. HIV NAAT results after birth), which are likely very important contributors to the NP microbiome. For example – a person with poorly controlled HIV and low CD4 count is at risk of opportunistic respiratory infections (TB, PJP, MAC) which would likely influence the composition of their NP microbiome. Was there really no information available to the study authors that could be included to better describe the health of the mothers and infants (any maternal viral loads or CD4 counts, known maternal comorbidities, infant dried blood spot testing at birth, etc.)?

Response 1-2: We agree that information on the immune status of the HIV+ mothers is indeed integral to identifying additional factors in the composition of their NP microbiota. We regrettably did not have access to this data as detailed HIV status information was not collected as part of the SAMIPS study, which was focused on Pertussis incidence. It was not optimally designed to look at the impact of HIV exposure as the original study purpose and did not seek to characterize the mothers HIV health status nor collect breastfeeding information. Neither viral load nor CD4 testing were routinely performed in Lusaka at this time and neither of these were deemed essential for the purposes of the original study beyond noting the mothers’ HIV status.

 We have clarified this point further in the Methods section on line 118, and in the discussion on lines 492-494 (“several key variables were not measured that would have been informative as potential confounders in our study results”).

Comment 1-3: Person-first language should be used throughout the abstract and manuscript. It is probably fine to use the abbreviations as-is (HIV+, HEU, HUU), but phrases such as “HIV-positive mothers” or “HIV-infected women” should be changed to “women/mothers with HIV” or “women/mothers living with HIV.” The term “mother-to-child transmission” is also outdated and should be changed to “vertical transmission” or “perinatal transmission.” For more information on why this matters, please see: Dilmitis *et al.* (2012 ^1^).

**References**

 1. Dilmitis S, Edwards O, Hull B, Margolese S, et al.: Language, identity and HIV: why do we keep talking about the responsible and responsive use of language? Language matters. *Journal of the International AIDS Society*. 2012; **15** (4(Suppl 2)). Publisher Full Text

Response 1-3: We thank the reviewer for their attentiveness to this matter. We have implemented person-first identifying language throughout the manuscript.

Comment 1-4: Introduction, paragraph 2: the authors reference “the HEU phenomenon” several times without really explaining what “the phenomenon” is. Are they referring to the increased morbidity and mortality observed in this population? Please be more specific.

Response 1-4: The reviewer’s inclination is correct; we are referring to the increased morbidity and mortality rates observed in HEU infants. We have altered the manuscript to read more clearly in the introduction, by removing one instance and clarifying in another case the “HEU ‘phenomenon’ of increased morbidity and/or mortality rates” (see lines 51, 64, 66).

 In lines 511-513 of the Conclusions, we altered a sentence reading “This warrants further research and discussion regarding the phenomenon” to “The effects we have found here warrant further research and discussion regarding the role HIV exposure plays in infant health before readily affirming or denying that HIV exposure affects infants’ NP microbiomes.”

Comment 1-5: Introduction, paragraph 4: The authors state that their study might “provide insight into the presence or absence of immunological factors distinguishing [HEU vs. HUU infants].” However, differences in the microbiome do not necessarily indicate differences in “immunological factors.” Recommend revising the text appropriately.

Response 1-5: We agree with the reviewer’s reasoning that differences present in the microbiome do not necessarily correlate to immunological differences. We made a jump in reasoning that can only be made with something akin to an analysis of host-microbe interactions, which was not conducted here. We have altered the sentence to read, “We reasoned that by characterizing quantifiable differences in NP microbiota distinguishing these two groups, this pilot study could indicate differences encouraging future immunology-focused studies” (lines 82-83).

Comment 1-6: Methods, paragraph 1: it is necessary to state whether all infants breastfed or whether some infants consumed any foods other than breast milk in the first 14 weeks of life, as nutrition sources have a profound ability to impact the microbiome.

Response 1-6: We agree that information on breastfeeding would be extremely relevant for the purposes of microbiome analysis. After reviewing the case report forms, we found that the study did not assess and collect breast feeding status. For purposes of this study, we can clarify that formula is rarely used and breastfeeding is nearly universal. We have clarified this in the manuscript and added a relevant citation (lines 123-125).

Comment 1-7: There are several examples where the manuscript is organized in a non-standard way:

 Tables containing data (such as Table 1 and Table 2) belong in the results section, not the methods section. Recommend moving these tables, and the text describing them, to the beginning of Results.

Response 1-7: We have moved the tables and appropriate text descriptions to the beginning of the Results section (lines 267-273).

Comment 1-8: The text in the methods section describing the reasons that some samples were excluded (beginning with “Some missingness in the data is present…”) also belongs in results, not methods.

Response 1-8: We have moved the description of the excluded samples to the beginning of the Results section (line 268).

Comment 1-9: There are some “editorial” comments in the results section, which really belong in the discussion instead. For example, page 7, last paragraph: “…which may in part be the result of the mothers’ acquired immunity over time and therefore lower overall NP carriage;” page 8, 3 ^rd^ to last paragraph: “These transitional patterns align with those found in a longitudinal East Asian infant cohort.”

Response 1-9: We have moved the comments in question to the Discussion section, including the larger paragraph on parallel findings from previous studies (lines 285-287; 421-422; other comments were also moved when relevant).

Comment 1-10: Table 1 is very confusing and poorly organized:

 The headers often do not apply to the rows beneath them (male sex and median age should not be categorized under “place of birth;” median birth weight and twin births should not be categorized under “gestational age;” number of mothers, mothers age, married, father lives with child should not be categorized under “immunizations at birth;” etc.

 There are many rows for which the result is 0 in every column, or for which there are apparently no data. These rows should be removed from the table completely. For example, why is the Chilenje clinic described in table when only the Chawama clinic is described in the text? Why is “other place of birth” listed in the table if none of the infants were born in an “other place”? Why are “father lives with child” and “trimester initiated ART” included in the table if there are no data for these variables? The L&D complications that did not occur in any infants (obstructed labor, sepsis, hemorrhage, other) can be removed and a sentence could be added to the text that says “none of these complications occurred: X,Y,Z.”

 The table shows one infant with birth asphyxia. This infant should be further described in the text. How severe was the birth asphyxia? Did the infant require respiratory support, intubation, oxygen, etc. (since respiratory instrumentation could potentially have impacted this infant’s respiratory microbiome)?

Response 1-10: We thank the reviewer for their attention to this oversight. We have reordered the rows to clearly define parameter sections, and removed all rows for which the parameter did not apply to the infant demographics.

 We have added the following sentence in place of the excluded rows: “None of the mothers in this cohort experienced obstructed labor, sepsis, or hemorrhage complications during the labor and delivery period” (lines 97-99).

 During the SAMIPS data collection procedures, each mother was asked whether they had any complications with labor and delivery. This was a closed ended question with five possible responses: obstructed labor, birth asphyxia, sepsis, hemorrhage, or other. Follow-up questions about severity or treatment were not asked. Given that the study eligibility criteria limited enrollment to healthy infants with no acute or chronic medical conditions, it is unlikely that the birth asphyxia was severe. However, we cannot rule out the use of respiratory instrumentation that could impact the infant’s respiratory microbiome. We have noted this clarification in our study limitations of the Discussion section (lines 502-503).

Comment 1-11: Table 2:

 The text states that two of 20 mothers lacked swabs at both the t=0 and t=6 time points. However, the table shows all 20 mothers had a swab at t=0 and 2 were missing swabs at t=6. Either the text or table should be revised so that they harmonize.

 It would be preferred if the table could be stratified by HIV status at each time point, rather than consolidating all of the HEU and HUU results at the bottom. This way it would be easy to see whether either group was over-represented at any one given time point.

Response 1-11: We appreciate the reviewer’s attention to this discrepancy. We verified that the table contains an accurate summary of the data and updated the text to reflect the table (line 273). We have also updated Table 2 to delineate each status/subject group per time point.

Comment 1-12: Table 3: please standardize the number of decimal points across columns (some columns have 2 decimal points, others have none).

Response 1-12: We have adjusted the table to have one decimal point standardized across the columns and right-justified the entries (now Table 7).

Comment 1-13: Table 4: several columns describe “adjusted p-values” but there is no description of what variables were adjusted for. The reader should not have to refer back to the methods section to understand the statistical analysis. This is also a problem in Table 7. Please add a description of what variables the analyses were adjusted for to the Table heading or to a footnote for each table.

Response 1-13: We thank the reviewer for their comment. We acknowledge that there may also be some confusion as to what we mean by adjusted p-values. The concept of adjusting p-values is widely used in ‘omics analysis, and stems from the application of a correction to limit the Type I error (false positive) inflation in multiple hypothesis testing. There are several techniques that can be used for this purpose; we used the Bonferroni correction which is the most conservative. The actual implementation of this correction is to identify a corrected significance level, α, so each p-value is compared to this new value resulting in an adjusted p-value.

 We have clarified language throughout the manuscript and caption for Table 7 (now Table 6) by changing to the following language: “A Bonferroni correction was applied to account for multiple hypothesis testing.” (lines 753-754).

 We recognize that Table 4 (now Table 3) is especially confusing given the different variables. We have rearranged the columns to list the coefficient estimate followed by the unadjusted and adjusted p-values for each variable included in the model.

Comment 1-14: Table 6: I believe there is a typo in the table; the two time points being compared are t=0 and t=6 according to the text; however, the table shows t=0 and t=1.

Response 1-14: We have adjusted Table 6 accordingly (now Table 5).

Comment 1-15: Table 7: It is very difficult to understand this table without reading the text. Specifically, what direction is indicated by the positive and negative numbers in the column titled “mean of differences” (higher abundance in infants or higher abundance in mothers)? After a long period of scrutiny, I was able to determine that negative numbers mean higher abundance in infants, but it should be immediately apparent to the reader. Please explain this in the Table heading or in a footnote.

Response 1-15: We agree that the mean of differences column is not well explained. We have gone ahead and corrected the table caption and heading to explain the sign directionality for mean of differences values for Table 7 (now Table 6).

Comment 1-16: Also related to Table 7, in the results section, page 12, paragraph 2: The authors describe that the purpose of the analysis in Table 7 is to compare the abundance of 3 particular bacterial species between the mothers and infants within each cohort (HIV+/HEU and HIV-/HUU), and that this comparison will help explain why HEU infants may be more susceptible to morbidity/mortality. It is not clear how comparing the abundance of these bacteria between mothers vs. infants WITHIN each cohort would help explain the HEU infants’ increased susceptibility to morbidity/mortality. It seems that to answer this question, it would be more valuable to compare the abundance of bacteria between HEU vs. HUU infants (or between HIV+ vs. HIV- mothers). Please clarify how you expect this analysis to answer the question proposed or modify/remove the language describing the reason for this analysis.

Response 1-16: We agree that the justification for performing this set of tests is invalid as written, and so we have rewritten the test justification as follows (see lines 363-366):

 “Although all infants in our cohort were free from illness, we found that certain pathogenic species were present in modest abundance across samples. To ascertain the possibility of HIV-exposed infants acquiring pathogens from their HIV(+) mothers, we conducted paired t-tests for three well-known pathogenic species: Streptococcus pneumoniae; Haemophilus influenzae; and Staphylococcus haemolyticus.”

Comment 1-17: Discussion: The authors should add to the list of limitations that their results may not be generalizable to HEU infants around the globe, given that all samples were collected from participants living in a single African country.

Response 1-17: We agree with this limitation and have added it to our conclusion accordingly on lines 490-491. (“Second, given that all samples were collected from participants born in Zambia, these results may not be generalizable to HIV-exposed infants in other countries.”)

Comment 1-18: Discussion, page 14: There is some redundancy between the last 2 sentences of the first paragraph, and the 6 ^th^ paragraph, both of which describe differences in bacterial diversity between HIV+ and HIV- mothers.

Response 1-18: We have relocated the sentence referring to Figure 1B (“From Figure 1B, we observed that…”) to lines 478-479 and removed the repetitive sentence referring to increased microbial diversity.

Comment 1-19: There are several typos in the manuscript. For example, page 11, paragraph 3: “The same test for all infant samples at time point six was contrastingly showed….” Also Conclusions: “our findings may suggest that that sociological….”

Response 1-19: We have made the appropriate corrections throughout the manuscript and thank the reviewer for their attention to this matter.
